# Cationic Heteroleptic Cyclometalated Iridium^III^ Complexes Containing Phenyl-Triazole and Triazole-Pyridine Clicked Ligands 

**DOI:** 10.3390/molecules15032039

**Published:** 2010-03-23

**Authors:** Marco Felici, Pablo Contreras-Carballada, Jan M. M. Smits, Roeland J. M. Nolte, René M. Williams, Luisa De Cola, Martin C. Feiters

**Affiliations:** 1 Department of Organic Chemistry, Institute for Molecules and Materials, Faculty of Science, Radboud University Nijmegen, Heyendaalseweg 135, 6525 AJ Nijmegen, The Netherlands; E-Mails: m.felici@science.ru.nl (M.F.); r.nolte@science.ru.nl (R.J.M.N.); 2 Van`t Hoff Institute for Molecular Sciences, Molecular Photonic Materials, Nieuwe Achtergracht 129, 1018 WS Amsterdam, The Netherlands; E-Mails: P.ContrerasCarballada@uva.nl (P.C.-C.); R.M.Williams@uva.nl (R.M.W.); 3 Solid State Chemistry, Institute for Molecules and Materials, Faculty of Science, Radboud University Nijmegen, Heyendaalseweg 135, 6525 AJ Nijmegen, The Netherlands; E-Mail: J.Smits@science.ru.nl (J.M.M.S.); 4 Physikalisches Institut, Westfälische Wilhelms-Universität Münster, Wilhelm-Klemm-Str. 10, 48149 Münster, Germany; E-Mail: decola@uni-muenster.de (L.D.C.)

**Keywords:** click chemistry, phenyl-triazole, pyridine-triazole, iridium, luminescence

## Abstract

Novel heteroleptic iridium complexes containing the 1-substituted-4-phenyl-1*H*-1,2,3-triazole (phtl) cyclometalating ligand have been synthesized. The 3+2 Huisgen dipolar cycloaddition method (‘click’ chemistry) was utilized to prepare a class of bidentate ligands (phtl) bearing different substituents on the triazole moiety. By using various ligands (phtl-R_1_ and pytl-R_2_) (R_1_=adamantane, methyl and R_2_=adamantane, methyl, β-cyclodextrin, ursodeoxycholic acid), we prepared a small library of new luminescent ionic iridium complexes [Ir(phtr-R_1_)_2_(pytl-R_2_)]Cl and report on their photophysical properties. The flexibility of the clicking approach allows a straightforward control on the chemical-physical properties of the complexes by varying the nature of the substituent on the ligand.

## 1. Introduction

**Scheme 1:**
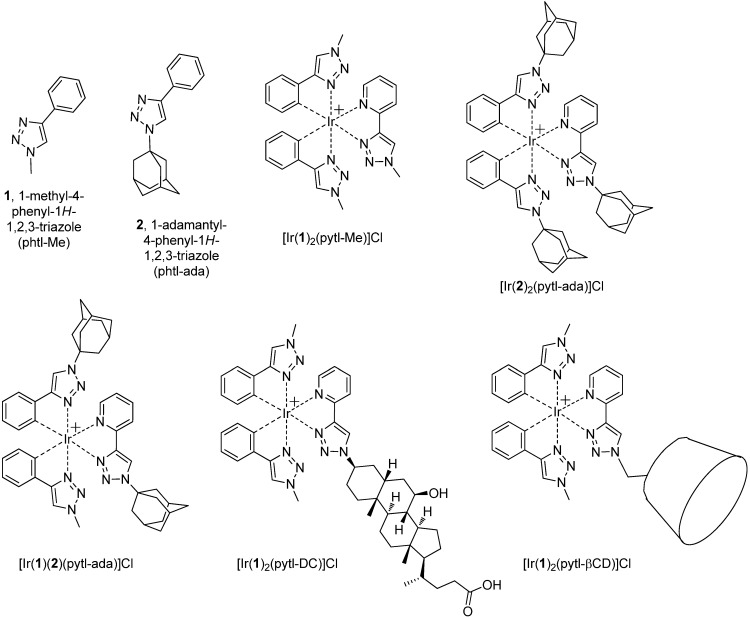
Structures of **1** and **2** and their heteroleptic iridium complexes.

The photoluminescence of organometallic complexes has attracted much interest since it can be utilized for a variety of applications such as oxygen sensors, biological probes and phosphorescent dopants in optoelectronic devices [1,2]. In particular, cyclometalating complexes of iridium^III^ have received great attention because of the high tunability of their emission in terms of color and efficiency. This has been achieved by screening a large variety of cyclometalating and ancillary ligands, often bearing functional groups with electron withdrawing or releasing properties. While much has been done on the investigation of the photophysical properties of iridium^III^ complexes, little attention has been focused on controlling other properties like solubility and polarity. The increasing number of applications of such compounds has made this aspect more appealing; it is indeed important, for example, to have photo-active materials which are water soluble in order to incorporate them in electrochemiluminescence (ECL) devices, or apolar with high affinity for hydrophobic matrices or with ‘sticky’ tails able to selectively recognize guests on modified surfaces [3]. The best approach to obtain complexes with designed physico-chemical properties is through the functionalization of ligands with selected substituents. Unfortunately, most of the ligands are difficult to prepare or their synthesis requires a large number of steps [4,5,6,7]. Their functionalization is not always trivial as it usually implies aromatic substitution reactions (on the phenyl or pyridyl moities) and coupling reactions. This all leads to an increase in the cost of the material itself and to a limited applicability. We now report on an exploration of the application of the Huisgen Cu-catalyzed [3+2] cycloaddition as an efficient and flexible means to prepare a class of bidentate cyclometalated ligands, 1-substituted-4-phenyl-1*H*-1,2,3-triazole (phtl) (**1** and **2**, Scheme 1) [8]; we have also investigated whether stable luminescent heteroleptic iridium complexes could be formed (Scheme 1), and what properties emerge from the use of such a 1,2,3-triazole-containing chelator. The substituents we used to functionalize pytl [2-(1-substituted-1*H*-1,2,3-triazol-4-yl)pyridine] and phtl [*i.e.,* βCD, adamantanes, ursodeoxycholic acid (UDC) derivative and methyl] are examples of moieties provided with different chemico-physical properties. Decoration of a metal complex with these residues does not influence the electronic properties of the molecule and thus it does not affect directly its correlated luminescent properties. However, it can add other properties to the metal complex providing us with multi-functional materials. We prepared a small library of phosphorescent complexes bearing either host (βCD) or guest molecules (adamantane, UDC-derivative). Cyclodextrins are well-known cyclic oligosaccharides that can form inclusion complexes in aqueous solution with a variety of hydrophobic substrates, such as adamantane derivatives, and have been widely applied as supramolecular building blocks in various areas [9,10,11,12,13,14], including photoactivated electron transfer processes [6,15,16,17,18]. With βCD attached to [Ir(**1**)_2_(pytl-βCD)]Cl the phosphorescent dye can be immobilized on guest-appended polymeric membranes, which are used as responsive materials for oxygen sensors. βCD can also act as second sphere ligand, enhancing the photophysical properties of [Ir(**1**)_2_(pytl-βCD)]Cl [19]. The presence of adamantanes in [Ir(**2**)_2_(pytl-ada)]Cl and [Ir(**1**)(**2**)(pytl-ada)]Cl allows the supramolecular decoration with iridium complexes of the surface of vesicles and nanoparticles covered with cyclodextrins [20]. Particularly interesting is the complex [Ir(**1**)_2_(pytl-DC)]Cl where a UDC derivative instead of adamantane was used as a host. Native UDC is a amphiphilic molecule which has high affinity for βCD [21] as well as for bilayer membranes [22,23,24,25]. As we show here, UDC can be easily functionalized through the azido group (Scheme 3). Its affinity for membranes, combined with the sensitivity of polyamine iridium^III^ complexes to the polarity of their environment, provides us with an interesting luminescent polarity probe for the study of the dynamics of natural and artificial membranes [4,5,26]. Besides the supramolecular aspects, the nature of the substituents on the complex [Ir(phtl-R_1_)_2_(pytl-R_2_)]Cl strongly affects other properties like solubility. This can be tuned by changing the hydrophilicity of the ligands: the simplest complex of the series, the fully methylated [Ir(**1**)_2_(pytl-Me)]Cl, displays a poor solubility in water which is dramatically increased in [Ir(**1**)_2_(pytl-βCD)]Cl by the introduction of one per-methylated βCD. 

## 2. Results and Discussion

### 2.1. Synthesis of phtl and pytl ligands and their iridium complexes

**Scheme 2:**
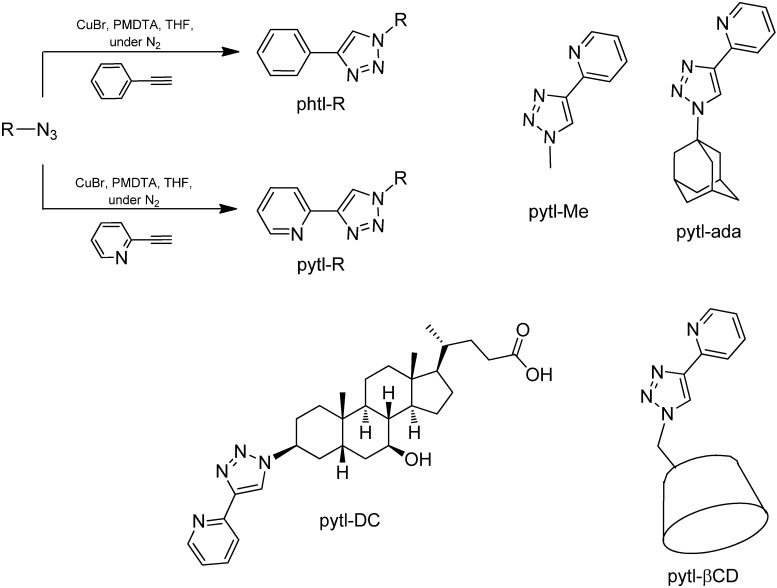
Synthesis and structures of the ligands phtl-R and pytl-R.

The Huisgen cycloaddition, also known as ‘click reaction’ [27], involves the formation of 1,2,3-triazole rings by coupling terminal alkynes and azides. It can be used to prepare the phtl ligand in one step, by reacting ethynylbenzene with an azide-containing molecule. The known high efficiency of this reaction, combined with its tolerance to other functional groups [27], allows the introduction of the phtl (and pytl) ligand on many different substrates (*i.e.* methyl, adamantane, bile acid) [19,28,29], requiring only the presence of at least one azido group on the molecule of interest. Therefore, this approach makes a large library of cyclometalating ligands readily accessible. The ligands **1** and **2** were prepared by reacting azidomethane and 1-azidoadamantane with ethynylbenzene for two hours in deoxygenated THF, in the presence of CuBr and pentamethyldiethylenetriamine (PMDTA). The products were isolated in 43% and 76% yield, respectively. The click reaction was used to prepare also the ancillary ligands pytl-R, by reacting 2-ethynylpyridine with the corresponding azido-appended derivatives in identical conditions (Scheme 2) as reported previously [19]. In addition to full characterization by NMR and mass spectrometry, the crystal structures of the ligands **1** and **2** were obtained by single crystal X-ray diffraction (Table S1, Figure S1 and S2). 

**Scheme 3:**
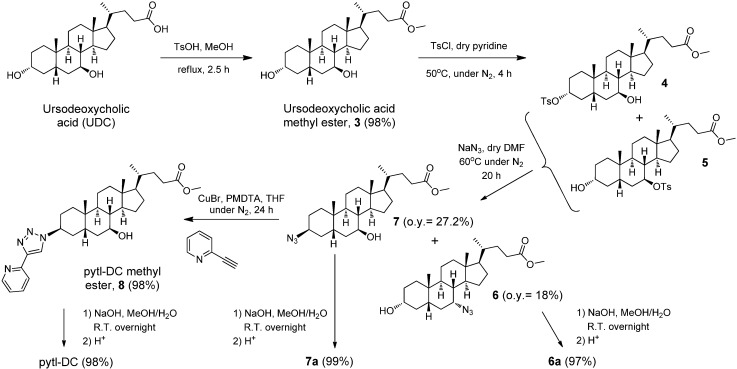
Synthesis of the cholic acid derivatives **6-8**.

The preparation of the ligand 3β-(4'-(pyridin-6'-yl)-1'*H*-1',2',3'-triazol-1'-yl)-7β-hydroxy-5β-cholan-24-oic acid (pytl-DC) required the synthesis of 3β-azido-7β-hydroxy-5β-cholan-24-oic acid methyl ester **7** (Scheme 3). This was achieved by adapting the procedure described in the literature for the azidation of cholic acid [30]. The carboxylic group was initially protected as methyl ester to avoid undesired reactions and the hydroxyl group converted into a better leaving group by tosylation. The latter step produces a mixture of tosylated derivatives that was further used without purification. The substitution of the tosylate by sodium azide accomplished a mixture of **6** and **7** separated by chromatography. Both compounds are very interesting for bio- and supramolecular applications. Due to the ability of the azido group to undergo different chemical modifications (click reaction, reduction to amino group), they can be appended to a wide range of substrates. 

**Figure 1:**
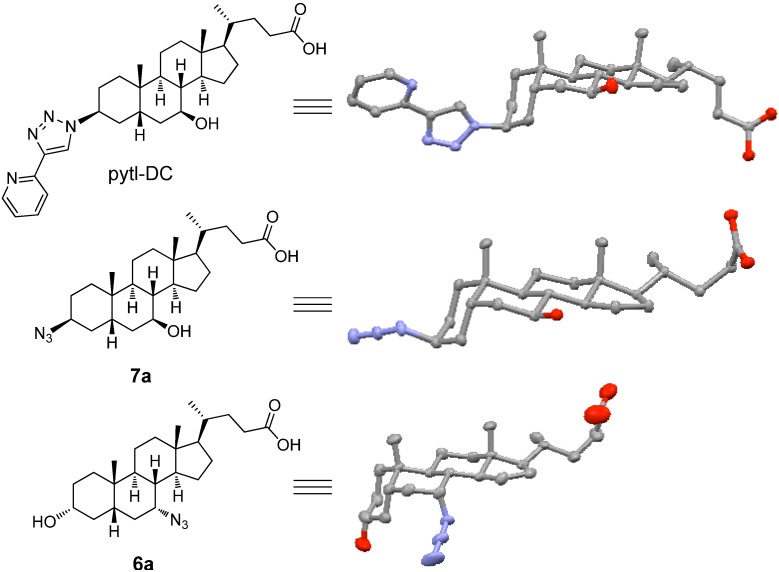
Crystal structures of pytl-DC, **6a** and **7a**. Gray, carbon; blue, nitrogen; red, oxygen. The thermal ellipsoids for the image represent 25% probability limit. Hydrogen atoms are omitted for clarity.

In addition to full characterization by NMR spectroscopy and mass spectrometry, the crystal structures of the carboxylic acids derivatives pytl-DC, **6a** and **7a** (Figure 1 and Table S2) were determined by single crystal X-Ray diffraction. It should be noted that the nucleophilic substitution of the tosyl group occurred with a bimolecular (S_N_2) mechanism resulting in the inverted stereochemistry of the carbon atom. This was proved by the crystal structures of **6a** and **7a** which clearly show that the azide is in an axial position (Figure 1). This aspect is particularly important in the case of **7a** because a derivative with a molecular rod-like shape results when the pytl moiety is attached (Figure 1). We note that, due to the inversion at the carbons 3 and 7 in ursodeoxycholic acid, **6a** and **7a** have in fact the configurations of chenodeoxycholic and isoursodeoxycholic acid, respectively, according to accepted deoxycholic acid nomenclature [31].

It is interesting to notice that the clicking approach allows the functionalization of phtl and pytl with substituents which display very different molecular complexity. We explored here the range from a simple methyl group to deoxycholic acid, which has a four-ring steroidal structure with two hydroxyl groups and one carboxylic acid functionality. The efficiency and high flexibility of such an approach provides a powerful tool for the preparation of large libraries of bidentate ligands.

**Scheme 4:**
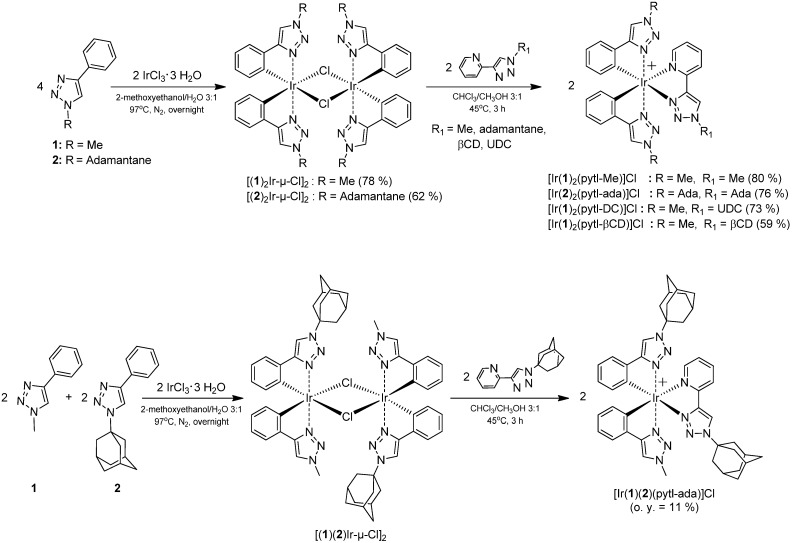
Synthesis of the iridium^III^ complexes.

The synthesis of an iridium^III^ complex is usually accomplished through a two-step process: the Nonoyama reaction that yields a chloride-bridged dinuclear iridium species [32] followed by the substitution of the chlorides with an ancillary ligand (Scheme 4). The iridium^III^ dimers [(**1**)_2_Ir-μ-Cl]_2_ and [(**2**)_2_Ir-μ-Cl]_2_ were prepared by reacting **1** or **2** with IrCl_3_, in a mixture water/2-methoxyethanol 1/3 (v/v) at 97 ºC. The compound [(**1**)(**2**)Ir-μ-Cl]_2_ was prepared by using an equimolar mixture of **1** and **2**, which afforded a mixture of dinuclear species difficult to separate and further reacted without purification. All the complexes were obtained by reacting the corresponding iridium dimer with a bidentate pytl ligand (Scheme 4) under mild conditions, in a chloroform/methanol 3:1 (v/v) mixture at 45 °C. After 3 hours the formation on TLC of only one new spot was observed, characterized (in contrast to the starting compound) by a bright luminescence under UV-Vis lamp at 366 nm. All the complexes, except for [Ir(**1**)(**2**)(pytl-ada)]Cl, were purified by silica column chromatography or preparative layer chromatography (PLC). In the case of [Ir(**1**)(**2**)(pytl-ada)]Cl, the non-selective synthesis resulted in a mixture of different iridium species which could not be isolated by traditional silica chromatography. We overcame this problem by using an HPLC apparatus equipped with a semi-preparative reversed-phase column. The characterization was done by means of NMR spectroscopy and high-resolution mass spectrometry. Compound [Ir(**1**)(**2**)(pytl-ada)]Cl is an example of cyclometalated complex where three different ligands are present: **1**, **2** and pytl-ada. It was synthesized to investigate the possibility to prepare complexes with a higher degree of functionalization. To our knowledge complexes with these structural characteristics have not been reported to date.

Cyclometalated iridium complexes have an octahedral geometry. The relative spatial orientation of the substituents around the metal center depends on whether they are attached to the cyclometalating or the ancillary ligands. In fact, it is known that in the chloride-bridged dinuclear iridium complex prepared with the Nonoyama reaction the cyclometalating ligands are always oriented in a trans position with respect to each other. In particular, the two nitrogens of the phtl ligands are aligned along a common axis together with the central iridium atom [33]. The pytl, which is inserted in the second synthetic step, is located on the plane perpendicular to that axis. By taking advantage of the well-established orientation of the ligands around the complex, considering that both phtl and pytl can be prepared starting from the same azide-appended molecule, we were able with our approach to control the location of the substituents in the iridium complex simply by clicking them to either 2-ethynylpyridine or ethynylbenzene.

### 2.2. Photophysical characterization

**Figure 2:**
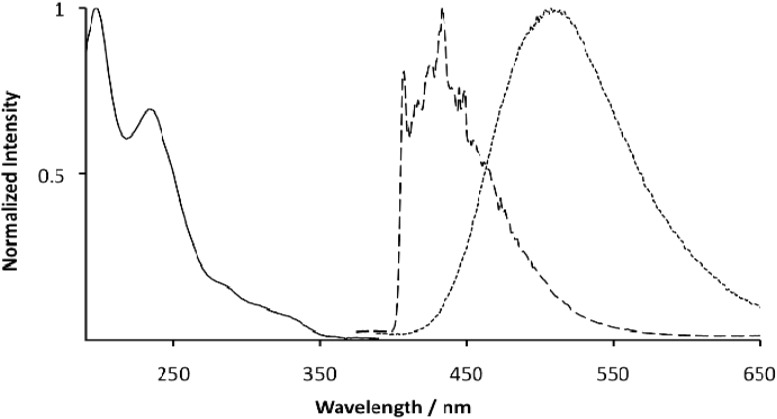
UV-Vis absorption spectrum (solid) and room temperature emission spectrum (dotted) in acetonitrile of [Ir(**1**)(**2**)(pytl-ada)]Cl (λ_exc_= 335 nm). 77K emission spectrum (dashed) in glassy butyronitrile matrix of [Ir(**1**)(**2**)(pytl-ada)]Cl (λ_exc_= 335 nm).

All the iridium complexes are soluble in acetonitrile and these solutions were used to measure the absorption and emission spectra. The UV-Vis spectra of all the complexes are very similar (Supplementary Data); we show here only the spectrum of the iridium complex [Ir(**1**)(**2**)(pytl-ada)]Cl (Figure 2). The intense absorption bands present in the UV region (190–240 nm) are assigned to ^1^(π-π*) transition of both phtl and pytl ligands (Figure 2). The shoulders appearing in the range between 290 and 350 nm are likely to be due to weaker charge transfer (CT) transitions, being of both spin-allowed ^1^MLCT and forbidden ^3^MLCT nature. All complexes are luminescent in solution at room temperature and display very similar, broad, featureless emission spectra between 410 and 650 nm (emission of [Ir(**1**)(**2**)(pytl-ada)]Cl, Figure 2) with a maximum for the emission at 510 nm. 

According to previously published work concerning analogous cyclometalated iridium^III^ complexes [34] such a broad emission is characteristic for a metal-to-ligand-charge-transfer excited state in which, formally speaking, an electron is transferred from the metal center to the ligand with the lowest reduction potential. If this transfer is understood as a HOMO-LUMO transition, the HOMO would be localized on the metal core and the LUMO orbital on the ligand. This is also consistent with the emission quantum efficiencies from all the complexes measured in air-equilibrated solutions which range from 0.021 to 0.052 (Table 1); in de-aerated conditions the compounds [Ir(**1**)_2_(pytl-Me)]Cl, [Ir(**2**)_2_(pytl-ada)]Cl and [Ir(**1**)(**2**)(pytl-ada)]Cl exibit a 2-3 fold increase of the emission quantum yields; however, for [Ir(**1**)_2_(pytl-DC)]Cl and [Ir(**1**)_2_(pytl-βCD)]Cl the increase is very large and reaches 5-fold. For [Ir(**1**)_2_(pytl-βCD)]Cl the quantum yields are remarkably high and comparable with the value measured for [Ir(ppy)_2_(pytl-ada)]Cl where the cyclometalating ligands were ppy [19]. This behaviour could be explained by the tendency of molecules like β-cyclodextrin and especially ursodeoxycholic acid to interact (aggregate or formation of inclusion complexes) even in very dilute solutions; this interaction could increase the emission efficiencies by reducing the non-radiative decay promoted by vibrational modes. Organization of organometallic complexes in solution through micelle or vesicle formation, inclusion in a cyclodextrin cavity or by other means has have been shown to influence greatly the properties of these complexes [35]. These changes in the emissive properties of the compounds studied are always assigned to changes in the surrounding. For example vesicle formation with metallosurfactants leads to the observation of fast components in the excited state decay of the individual metal complexes [36]. All these complexes undergo large hypsochromic shifts upon cooling to 77 K (spectrum of [Ir(**1**)(**2**)(pytl-ada)]Cl, Figure 2). In a butyronitrile solid matrix the maxima for the emission are blue-shifted and appear at 435 nm. Such a behavior is expected for compounds emitting from a charge transfer excited state and it is due to the impossibility for the solvent molecules to rearrange around the excited state. The consequent lack of stabilization of the excited state leads to a more energetic (blue-shifted) emission. The emission spectra show a series of vibronic transitions (emission of [Ir(**1**)(**2**)(pytl-ada)]Cl, Figure 2) which are not resolved at room temperature and which imply that a considerable ligand-centered (LC) character develops in these species at low temperature [37].

**Table 1:** Luminescence lifetimes and quantum yields of emission of the complexes. For quantum yield measurements λ_exc_= 335 nm. For lifetime measurements λ_exc_= 324 nm. The solutions were measured in air equilibrated acetonitrile (air) and argon saturated (Ar) for degassing by bubbling argon for 20-30 minutes through the solution.

Complex	Φ(air)	Φ(Ar)	τ (ns, air)	τ (ns, Ar)
[Ir(**1**)_2_(pytl-Me)]Cl	0.037	0.097	24.8	27.1
[Ir(**2**)_2_(pytl-ada)]Cl	0.021	0.048	17.4	18.0
[Ir(**1**)(**2**)(pytl-ada)]Cl	0.024	0.071	11.8 (60%)	19.7
			23.6 (40%)	
[Ir(**1**)_2_(pytl-DC)]Cl	0.039	0.203	24	28.3 (84%)
				2.22 (16%)
[Ir(**1**)_2_(pytl-βCD)]Cl	0.052	0.254	26.4	34.1

## 3. Conclusions

In conclusion, we report the preparation and the photophysical properties of new highly luminescent heteroleptic iridium^III^ complexes. Click chemistry was used as an efficient tool to introduce on the phtl and pytl ligands functional groups provided with various level of complexity. The flexibility of such approach provides access to a large library of clicked ligands and therefore to multi-functional iridium^III^ complexes with tunable chemico-physical properties. In particular the complex [Ir(**1**)_2_(pytl-DC)]Cl is a promising luminescent polarity probe for the study of membrane dynamics; [Ir(**1**)_2_(pytl-βCD)]Cl and [Ir(**2**)_2_(pytl-ada)]Cl are interesting building blocks for supramolecular systems. Here we also report the first synthesis of the 3β-azido-7β-hydroxy-5β-cholan-24-oic acid (**7a**) and the isomer 3α-hydroxy-7α-azido-5β-cholan-24-oic (**6a**), both interesting intermediates for both bio- and supramolecular systems.

## 4. Experimental

### 4.1. General

THF was purified by distillation under nitrogen from sodium/benzophenone and dry DMF was purchased from Fluka. All other chemicals were purchased from Aldrich, Fluka or Acros and used as received. Analytical thin layer chromatography (TLC) was performed on Merck precoated silica gel 60 F-254 plates (layer thickness 0.25 mm) and the compounds visualized by ultraviolet (UV) irradiation at λ = 254 nm and/or λ = 366 nm and by staining with phosphomolybdic acid reagent or KMnO_4_. Preparative layer chromatography (PLC) was performed on Merck precoated silica gel 60 F-254 plates (layer thickness 1 mm, concentrating zone 20 × 4 cm) and the compounds visualized by ultraviolet (UV) irradiation at λ = 254 nm and/or λ = 366 nm. Purifications by silica gel chromatography were performed using Acros (0.035 – 0.070 mm, pore diameter ca. 6 nm) silica gel. All click reactions were performed in oxygen-free atmosphere of N_2_ using Schlenk conditions and solvents dried and distilled according to standard laboratory procedures.

*Nuclear magnetic resonance (NMR)*: ^1^H-NMR spectra were recorded, at 25 °C, on a Varian Inova 400 or a Bruker DMX-300 machines operating at 400 and 300 MHz, respectively. ^13^C-NMR spectra were recorded on a Bruker DMX-300 machine operating at 75 MHz. ^1^H-NMR chemical shifts (δ) are reported in parts per million (ppm) relative to a residual proton peak of the solvent, δ = 3.31 for CD_3_OD, δ = 7.26 for CDCl_3_ and δ = 2.50 for DMSO. Multiplicities are reported as: s (singlet), d (doublet), t (triplet), q (quartet), dd (doublet of doublets), ddd (doublet of doublet of doublets) or m (multiplet). Broad peaks are indicated by b. Coupling constants are reported as a J value in Hertz (Hz). The number of protons (n) for a given resonance is indicated as nH, and is based on spectral integration values. ^13^C-NMR chemical shifts (δ) are reported in ppm relative to a carbon peak of the solvent, δ = 49.0 for CD_3_OD, δ = 77 for CDCl_3_ and δ = 40 for DMSO. The signals of the protons on hydroxyl and carboxylic groups could not be observed due to the fast exchange with traces of water present in the deuterated solvents. The resolution of ^13^C spectra was increased when necessary by performing an exponential apodization of the FID. 

*Mass spectrometry (MS)*: All the mass analysis were performed by using electrospray techniques (ESI). High-Resolution mass spectrometry measurements were performed on a JEOL AccuTOF instrument by using water, acetonitrile or methanol as solvents. Standard mass spectrometry measurements were performed on a FINNIGAN LCQ Advantage Max by using water, acetonitrile or methanol as solvents.

*High-performance liquid chromatography (HPLC)*: High-performance liquid chromatography (HPLC) was carried out on a Shimadzu LC-20AT HPLC system equipped with a UV-Vis detector SPD-10AV and a fraction collector. Columns were purchased from Dr. Maisch GmbH. The compounds were purified on mg scale using a semipreparative reversed-phase HPLC column. A 2 mL solution was injected in a column ReproSil 100 C8, 5 μ (250X10 mm) operating at 30 °C. The detection wavelengths were fixed at 254 and 215 nm. A gradient of H_2_O and acetonitrile both containing either 0.1% v/v HCl or TFA was used as mobile phase, with a flow rate of 4 mL min^-1^. HCl was used to ensure that the chloride was the only counterion of the isolated compounds. In all cases the samples were prepared by dissolving the compound in mixtures H_2_O/acetonitrile 95/5 or 1/1 v/v, and filtered on a nylon syringe filter (0.2 μm). 

*X-ray crystallography*: Single crystals of **1** (phtl-Me) and **2** (phtl-ada) were grown by slow evaporation of a solution of compounds 1 and 2 in CHCl_3_/heptane. Single crystals of pytl-DC were grown by slow evaporation of a solution of pytl-DC in CH_3_OH. Single crystals of **6a** and **7a** were grown by slow evaporation of a slightly acidic solution of **6a** and **7a** in H_2_O/acetonitrile. The crystal data and summaries of the data collection and structure refinement are given in Table S1 for compounds **1** and **2**, and Table S2 for **6a**, **7a**, and pytl-DC; selected distances and bond angles as well as atomic coordinates and equivalent isotropic displacement parameters for the non-hydrogen atoms are also given in the Supplementary Information. All measurements were performed at -65 °C. The structures of **1**, **6a**, and **7a** were solved by the program SHELXS [38], those of **2** and pytl-DC by the program CRUNCH [39]. All non-hydrogen atoms were refined with anisotropic temperature factors. The hydrogen atoms were placed at calculated positions, and refined isotropically in riding mode. Crystallographic data (excluding structure factors) for the structures reported in this paper have been deposited with the Cambridge Crystallographic Data Centre as supplementary publication CCDC-1003/, deposition codes: **1** (FELIC3), 757888; **2** (MFR130), 757886; **6a** (MFR99), 757884; **7a** (MFR99B), 757885; pytl-DC (PYTLUD), 757887. Copies of available material can be obtained, free of charge, on application to the Director, CCDC, 12 Union Road, Cambridge CB2 1EZ, UK, (fax: +44-(0) 1223-336033 or e-mail:teched@chemcrys.cam.ac.uk).

*Emission and UV-vis*: Electronic absorption spectra were recorded in a quartz cuvette (1 cm, Hellma) on Hewlett-Packard 8543 diode array spectrometer (range 190 nm-1100 nm). Steady state fluorescence spectra were recorded using a Spex 1681 fluorimeter, equipped with a Xe arc light source, a Hamamatsu R928 photomultiplier tube detector and double excitation and emission monochromator. Emission spectra were corrected for source intensity and detector response by standard correction curves, unless otherwise noted. Luminescence quantum yields (Φ_em_) were measured in optically dilute solutions (O.D. < 0.1 at excitation wavelength), using [Ru(bpy)_3_]Cl_2_ in aerated and deoxygenated H_2_O (Φ_em, air_ = 0.028; Φ_em, deox_ = 0.042) or diphenylanthracene in aerated and deoxygenated cyclohexane (Φ_em, air_ = 0.77; Φ_em, deox_ = 0.91) as references. 

Lifetimes of excited states were determined using a coherent Infinity Nd:YAG-XPO laser (2 ns pulses fwhm) and a Hamamatsu C5680-21 streak camera equipped with Hamamatsu M5677 low speed single sweep unit. Streak cameras are high-speed light detectors, which enable detection of the fluorescence as a function of the spectral and the time evolution simultaneously. 

### 4.2. Synthesis and characterization

*1-Methyl-4-phenyl-1H-1,2,3-triazole* (**1**): Dry DMF was deoxygenated prior to use by performing three freeze-pump-thaw cycles. A two-necked round bottom flask was carefully dried with flame, under N_2_ flow. In the cooled flask, NaN_3_ (198 mg, 3 mmol) was added together with deoxygenated dry DMF (40 mL). CH_3_I (0.3 mL, 4.8 mmol) was added dropwise and the solution was stirred in the dark overnight. The methyl-azide formed is a highly explosive intermediate and therefore it was not isolated [40]. The ‘clicking’ reagents were subsequently added to the reaction mixture and a large excess of ethynylbenzene (658.9 μL, 6 mmol) and of catalyst were used to ensure the complete consumption of the methyl-azide. A solution of CuBr (430 mg, 3 mmol) and PMDTA (0.67 mL, 3.2 mmol) was prepared by dissolving both reagents in oxygen-free dry DMF (10 mL), bubbling nitrogen through the solution for 20 min to prevent oxidation of Cu(I). When the CuPMDTA complex dissolved, an aliquot of the solution (5 mL), together with compound ethynylbenzene (0.45 mL, 4.5 mmol) were added to the flask. The resulting mixture was stirred in the dark at room temperature under a nitrogen atmosphere for 24 hours. The reaction was followed by TLC (eluent: Et_2_O). After removal of the solvent *in vacuo* (CAUTION: this solution may still contain methyl azide in case the conversion with phenylacetylene was not 100 %) the solid obtained was purified by column chromatography (Et_2_O/Heptane 50/50 followed by Et_2_O/ethyl acetate 90/10). Product **1** was obtained as a white solid (208 mg, overall yield 43%). Crystals were grown by slow evaporation of a solution of **1** in CHCl_3_/heptane. The structure was further confirmed by X-ray single crystal diffraction. ^1^H-NMR (300 MHz, CDCl_3_): δ ppm 7.86–7.78 (m, 2H), 7.73 (s, 1H), 7.47–7.38 (m, 2H), 7.37–7.29 (m, 1H), 4.14 (s, 3H); ^13^C-NMR (75 MHz, CDCl_3_): δ ppm 148.0, 130.6, 128.8, 128.1, 125.7, 120.5, 36.7; HRMS (ES+, CHCl_3_/CH_3_OH): m/z calcd for C_9_H_10_N_3_: 160.08747; found: 160.08768 [M+H]^+^.

*1-Adamantyl-4-phenyl-1H-1,2,3-triazole* (**2**): Distilled THF was bubbled with N_2_ for 1h prior to use in order to remove the oxygen. Ethynylbenzene (185.6 μL, 1.69 mmol) and 1-adamantylazide (300 mg, 1.69 mmol) were added under nitrogen atmosphere in a Schlenk tube and dissolved in deoxygenated THF (15 mL). A solution of CuBr (242.4 mg, 1.69 mmol) and PMDTA (365.8 μL, 1.75 mmol) was prepared by dissolving both reagents in deoxygenated THF (15 mL) and bubbling nitrogen through the solution for 20 min to prevent oxidation of Cu(I). When the CuPMDTA complex dissolved, the solution became slightly green, and an aliquot of it (3 mL) was added to the Schlenk tube. The resulting mixture was stirred in the dark, at room temperature under a nitrogen atmosphere for 2.5 hours. The reaction was followed by TLC (eluent: Et_2_O/heptane 80/20). No workup was done and after removal of the solvent *in vacuo*, the blue-green solid obtained was directly purified by column chromatography (Et_2_O/heptane 30/70). Product **2** was obtained as a white solid (358.8 mg, 76%). Crystals were grown by slow evaporation of a solution of **2** in CHCl_3_/heptane. The structure was further confirmed by X-ray single crystal diffraction. ^1^H-NMR (300 MHz, CDCl_3_): δ ppm 7.86–7.81 (m, 2H), 7.82 (s, 1H), 7.45–7.38 (m, 2H), 7.34–7.28 (m, 1H), 2.32–2.26 (m, 9H), 1.85–1.79 (m, 6H); ^13^C-NMR (75 MHz, CDCl_3_): δ ppm 137.8, 131.1, 128.8, 127.8, 125.6, 118.2, 59.6, 43.0, 35.9, 29.5; HRMS (ES+, CHCl_3_/CH_3_OH): m/z calcd for C_18_H_22_N_3_: 280.18137; found: 280.18165 [M+H]^+^.

*3α,7β-Dihydroxy-5β-cholan-24-oic acid methyl ester* (**3**): Ursodeoxycholic acid (UDC, 277.1 mg, 0.71 mmol) was dissolved in methanol (10 mL). A catalytic amount of p-toluensolfonic acid was added and the solution was stirred under reflux for 2.5 hours. The reaction was followed by TLC (eluent: ethyl acetate/heptane 80/20). After removal of the solvent *in vacuo*, the crude product was dissolved in CHCl_3_ and washed with aqueous solution of K_2_CO_3_ (1M, 1 × 100 mL). The organic phase was dried over Na_2_SO_4_ anhydrous and the solvent removed under reduced pressure to yield **3** as a white solid (282.3 mg, 98%). ^1^H-NMR (300 MHz, CDCl_3_): δ ppm 3.64 (s, 3H), 3.61–3.48 (m, 2H), 2.41–2.10 (m, 2H), 2.01–0.96 (m, 24H), 0.92 (s, 3H), 0.90 (d, *J* = 6.4 Hz, 3H), 0.65 (s, 3H); ^13^C-NMR (75 MHz, CDCl_3_): δ ppm 174.7, 71.3, 71.2, 55.7, 54.9, 51.4, 43.69, 43.66, 42.4, 40.1, 39.2, 37.3, 36.9, 35.2, 34.9, 34.0, 31.01, 30.96, 30.3, 28.5, 26.8, 23.3, 21.1, 18.3, 12.1.

*Mixture of 3α-(4-Methylphenyl)sulfonyloxy-7β-hydroxy-5β-cholan-24-oic acid methyl ester* (**4**) *and 3α-hydroxy-7β-(4-Methylphenyl)sulfonyloxy-5β-cholan-24-oic acid methyl ester* (**5**): Compound **3** (254.5 mg, 0.62 mmol) was added under nitrogen atmosphere in a Schlenk tube and dissolved in dry pyridine (5 mL). Tosyl chloride (178.4 mg, 0.94 mmol) was added and the solution was heated to 50 ºC under nitrogen for 4 hours. The reaction was followed by TLC (eluent: ethyl acetate/heptane 50/50). After removal of the solvent in vacuo, the crude product was dissolved in ethyl acetate, washed with HCl (1N, 3 × 80 mL) and water (1 × 80 mL). The organic phase was dried over Na_2_SO_4_ anhydrous and the solvent removed under reduced pressure. The crude product (348.4 mg) was further reacted without purification.

*3α-Hydroxy-7α-azido-5β-cholan-24-oic acid methyl ester* (**6**) *and 3β-azido-7β-hydroxy-5β-cholan-24-oic acid methyl ester* (**7**): The crude material containing compounds **4**+**5** (348.4 mg) was added under nitrogen atmosphere in a Schlenk tube and dissolved in dry DMF (15 mL). Sodium azide (201.5 mg, 3.1 mmol) was added and the solution was heated to 60 ºC under nitrogen for 20 hours. The reaction was followed by TLC (eluent: ethyl acetate/heptane 50/50). After removal of most of the solvent *in vacuo*, the crude material was dissolved in ethyl acetate and washed with water (2 × 80 mL). The organic phase was dried over Na_2_SO_4_ anhydrous and the solvent removed under reduced pressure. The crude material was purified by column chromatography (eluent: ethyl acetate/heptane 15/85). Product **6** was obtained as a slightly yellow viscous oil (48.2 mg, overall yield = 18%). Product **7** was obtained as a slightly yellow viscous oil (72.8 mg, overall yield = 27.2%).

**6**: ^1^H-NMR (300 MHz, CDCl_3_): δ ppm 3.73–3.68 (m, 1H), 3.66 (s, 3H), 3.55–3.43 (m, 1H), 2.43–0.95 (m, 26H), 0.92 (d, *J* = 6.1 Hz, 3H), 0.91 (s, 3H), 0.63 (s, 3H); ^13^C-NMR (75 MHz, CDCl_3_): δ ppm 174.7, 71.8, 60.5, 55.6, 51.5, 51.1, 42.6, 41.0, 39.3, 38.3, 38.2, 35.3, 35.2, 34.9, 33.7, 31.0, 30.9, 30.6, 30.4, 28.0, 23.3, 22.9, 20.4, 18.3, 11.7. 

**7**: ^1^H-NMR (300 MHz, CDCl_3_): δ ppm 3.93–3.88 (m, 1H), 3.66 (s, 3H), 3.59–3.46 (m, 1H), 2.41–2.15 (m, 2H), 2.04–0.99 (m, 24H), 0.97 (s, 3H), 0.92 (d, *J* = 6.4 Hz, 3H), 0.67 (s, 3H); ^13^C-NMR (75 MHz, CDCl_3_): δ ppm 174.6, 71.3, 58.3, 55.8, 54.9, 51.5, 43.7, 43.6, 40.1, 38.9, 37.8, 36.3, 35.2, 34.4, 31.2, 31.01, 30.98, 30.3, 28.5, 26.8, 24.5, 23.7, 21.3, 18.3, 12.1.

*3α-Hydroxy-7α-azido-5β-cholan-24-oic acid* (**6a**) and *3β-azido-7β-hydroxy-5β-cholan-24-oic acid* (**7a**): A solution of NaOH was prepared by adding an aqueous solution of NaOH (2 N, 2 mL) into CH_3_OH (7 mL). **6a** (20 mg, 0.046 mmol) or **7a** (20 mg, 0.046 mmol) were dissolved in this solution and the resulting mixture was stirred at room temperature overnight. The reaction was followed by TLC (eluent: ethyl acetate). While cooling the reaction in ice bath, HCl (4 N) was added until pH = 7. After removal of the solvent *in vacuo*, the crude material obtained was purified by column chromatography (eluent: ethyl acetate). Product **6a** was obtained as a white solid (18.6 mg, 97%). Product **7a** was obtained as a white solid (19 mg, 99%). Single crystals of **6a** and **7a** were grown by slow evaporation of their slightly acidic solution in H_2_O/acetonitrile. The structures were further confirmed by X-ray single crystal diffraction. Alternatively, compounds **6a** and **7a** could be isolated from the crude mixture by semipreparative HPLC equipped with a reversed-phase column. The mobile phase was a gradient of H_2_O and acetonitrile (30% to 0% v/v of H_2_O in 35 min) both containing 0.1% v/v TFA, with a flow rate of 4 mL min^-1^.

**6a**: ^1^H-NMR (300 MHz, CDCl_3_): δ ppm 3.73–3.68 (m, 1H), 3.56–3.44 (m, 1H), 2.46–0.95 (m, 26H), 0.95–0.90 (m, 6H), 0.64 (s, 3H); ^13^C-NMR (75 MHz, CDCl_3_): δ ppm 179.0, 71.8, 60.4, 55.6, 51.1, 42.6, 41.0, 39.3, 38.3, 38.1, 35.3, 35.2, 34.9, 33.7, 30.8, 30.7, 30.6, 30.4, 28.0, 23.6, 22.9, 20.5, 18.2, 11.8.

**7a**: ^1^H-NMR (300 MHz, CDCl_3_): δ ppm 3.93–3.89 (m, 1H), 3.57–3.45 (m, 1H), 2.45–2.22 (m, 2H), 2.04–1.0 (m, 24H), 0.98 (s, 3H), 0.94 (d, *J* = 6.4 Hz, 3H), 0.69 (s, 3H); ^13^C-NMR (75 MHz, CDCl_3_): δ ppm 178.8, 71.4, 58.3, 55.8, 54.9, 43.8, 43.6, 40.1, 38.9, 37.9, 36.3, 35.2, 34.5, 31.3, 30.8, 30.3, 29.7, 28.6, 26.8, 24.6, 23.8, 21.4, 18.4, 12.1.

**Scheme 5:**
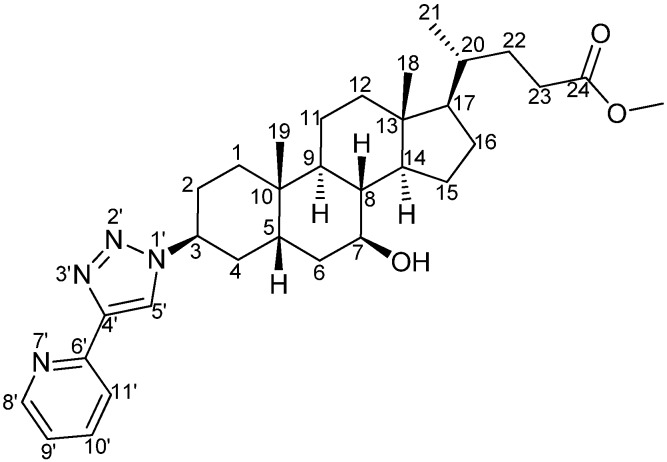
Structure and atom labeling of **8**.

*3β-(4'-(Pyridin-6'-yl)-1'H-1',2',3'-triazol-1'-yl)-7β-hydroxy-5β-cholan-24-oic acid methyl ester* (**8**) (Scheme 5): Distilled THF was bubbled with N_2_ for 1 hour prior to use in order to remove the oxygen. 2-Ethynylpyridine (58.6 μL, 0.58 mmol) and **7** (250 mg, 0.58 mmol) were added under nitrogen atmosphere in a Schlenk tube and dissolved in deoxygenated THF (10 mL). A solution of CuBr (83.2 mg, 0.58 mmol) and PMDTA (125.4 μL, 0.6 mmol) was prepared by dissolving both reagents in deoxygenated THF (10 mL) and bubbling nitrogen through the solution for 20 min to prevent oxidation of Cu(I). When the CuPMDTA complex dissolved, the solution became slightly green, and an aliquot of it (4 mL) was added to the Schlenk tube. The resulting mixture was stirred in the dark, at room temperature under a nitrogen atmosphere for 24 hours. The reaction was followed by TLC (eluent: ethyl acetate). After removal of the solvent *in vacuo*, the blue-green solid obtained was directly purified by column chromatography (eluent: ethyl acetate/heptane 50/50 followed by ethyl acetate/heptane 60/40). Product **8** was obtained as a white solid (303.9 mg, 98%).^ 1^H-NMR (400 MHz, CDCl_3_): δ ppm 8.57 (ddd, *J* = 4.9, 1.7, 0.9 Hz, 1H), 8.24 (s, 1H), 8.20 (ddd, *J* = 8.0, 1.0, 1.0, Hz, 1H), 7.77 (ddd, *J* = 7.8, 7.8, 1.8 Hz, 1H), 7.22 (ddd, *J* = 7.6, 4.9, 1.1 Hz, 1H), 4.72–4.68 (m, 1H), 3.66 (s, 3H), 3.67–3.57 (m, 1H), 2.40–1.02 (m, 26H), 0.94–0.91 (m, 6H), 0.68 (s, 3H); ^13^C-NMR (75 MHz, CDCl_3_): δ ppm 174.5, 150.3, 149.1, 147.6, 136.8, 122.6, 121.0, 120.1, 70.9, 56.5, 55.8, 54.8, 51.3, 43.5, 43.2, 39.9, 39.4, 37.6, 36.2, 35.1, 34.2, 30.94–30.77 (m), 30.4, 28.4, 26.7, 24.8, 23.4, 21.3, 18.2, 12.0; HRMS (ES+, CH_3_OH): m/z calcd for C_32_H_46_N_4_NaO_3_: 557.34676; found: 557.34578 [M+Na]^+^.

**Scheme 6:**
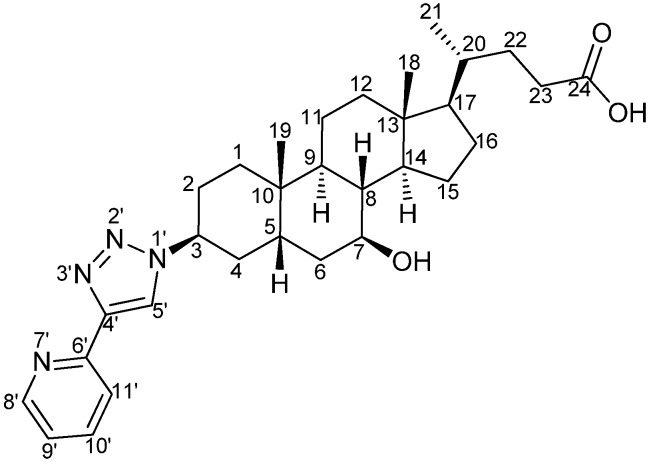
Structure and atom labeling of pytl-DC.

*3β-(4'-(Pyridin-6'-yl)-1'H-1',2',3'-triazol-1'-yl)-7β-hydroxy-5β-cholan-24-oic acid* (pytl-DC) (Scheme 6): A solution of NaOH was prepared by adding an aqueous solution of NaOH (2 N, 2 mL) into CH_3_OH (7 mL). **8** (55.9 mg, 0.10 mmol) was dissolved in this solution and the resulting mixture was stirred at room temperature overnight. The reaction was followed by TLC (eluent: CH_3_OH/ethyl acetate 10/90). While cooling the reaction in ice bath, HCl (4 N) was added until pH=7–8. After removal of the solvent *in vacuo*, the crude material obtained was purified by column chromatography (CH_3_OH/ethyl acetate 10/90). Pytl-DC was obtained as a white solid (55.6 mg, 98%). Crystals were grown by slow evaporation of a solution of pytl-DC in CH_3_OH. The structure was further confirmed by X-ray single crystal diffraction. ^1^H-NMR (300 MHz, DMSO-d_6_): δ ppm 8.67 (s, 1H), 8.59 (ddd, *J* = 4.9, 1.8, 1.0 Hz, 1H), 8.05 (ddd, *J* = 8.0, 1.1, 1.1 Hz, 1H), 7.89 (ddd, *J* = 8.0, 7.8, 1.8 Hz, 1H), 7.33 (ddd, *J* = 7.7, 4.9, 1.1 Hz, 1H), 4.74–4.68 (m, 1H), 3.44–3.32 (m, 1H), 2.29–0.93 (m, 26H), 0.89 (d, *J* = 6.4 Hz, 3H), 0.85 (s, 3H), 0.63 (s, 3H); ^13^C-NMR (75 MHz, DMSO-d_6_): δ ppm 175.8, 150.7, 149.9, 147.4, 137.6, 123.3, 122.9, 119.9, 69.8, 56.6, 56.2, 55.3, 43.5, 43.2, 40.2, 39.3, 38.1, 37.2, 35.3, 34.4, 31.6, 31.4, 31.0, 30.7, 28.6, 27.1, 24.8, 24.0, 21.6, 18.8, 12.5; HRMS (ES+, CH_3_OH): m/z calcd for C_31_H_44_N_4_NaO_3_: 543.33111; found: 543.33058 [M+Na]^+^.

*[(**1**)_2_Ir-µ-Cl]_2_*: A mixture 3/1 of 2-methoxyethanol/water (9 mL) was deoxygenated by bubbling nitrogen through it for 10-15 min. The ligand **1** (91.5 mg, 0.57 mmol) and IrCl_3_·3H_2_O (70 mg, 0.23 mmol) were added and the solution was heated to 97 ºC in the dark for 24 hours, under nitrogen atmosphere (without bubbling). After cooling to room temperature, the yellow solid was filtered off, washed with water (3 × 100 mL), diethyl ether (3 × 100 mL) and dried. After purification by column chromatography (eluent: from MeOH/CHCl_3_ 5/95 to MeOH/CHCl_3_ 10/90), the chloride-bridged complex [(**1**)_2_Ir-µ-Cl]_2_ was obtained as a yellow solid (97.4 mg, 78%). ^1^H-NMR (400 MHz, DMSO-d_6_): δ ppm 8.61 (s, 2H), 8.59 (s, 2H), 7.42 (ddd, *J* = 7.4, 1.4, 0.5 Hz, 2H), 7.41 (ddd, *J* = 7.5, 1.4, 0.5 Hz, 2H), 6.81 (ddd, *J* = 7.4, 7.4, 1.2 Hz, 2H), 6.80 (ddd, *J* = 7.4, 7.4, 1.2 Hz, 2H), 6.64 (ddd, *J* = 7.4, 7.4, 1.4 Hz, 2H), 6.63 (ddd, *J* = 7.4, 7.4, 1.5 Hz, 2H), 6.05 (ddd, *J* = 7.6, 1.2, 0.5 Hz, 2H), 6.00 (ddd, *J* = 7.6, 1.2, 0.5 Hz, 2H), 4.30–4.26 (m, 12H). ESI-MS (ES+, CHCl_3_/CH_3_OH): *m/z* = 1053.1 [M-Cl]^+^.

*[(**2**)_2_Ir-µ-Cl]_2_*: A mixture 3/1 of 2-methoxyethanol/water (9 mL) was deoxygenated by bubbling nitrogen through it for 10-15 min. The ligand **2** (50 mg, 0.18 mmol) and IrCl_3_⊕3H_2_O (21.4 mg, 0.072 mmol) were added and the solution was heated to 97 ºC in the dark for 24 hours, under nitrogen atmosphere (without bubbling). After cooling to room temperature, the yellow solid was filtered off, washed with water (3 × 80 mL) and dried. After purification by column chromatography (eluent: ethyl acetate), the chloride-bridged complex [(**2**)_2_Ir-µ-Cl]_2_ was obtained as a yellow solid (35 mg, 62%). ^1^H- NMR (400 MHz, CDCl_3_): δ ppm 7.83 (s, 2H), 7.77 (s, 2H), 7.27–7.22 (m, 4H), 6.75 (ddd, *J* = 7.4, 7.4, 1.1 Hz, 2H), 6.70 (ddd, *J* = 7.3, 7.3, 1.2 Hz, 2H), 6.64 (ddd, *J* = 7.2, 7.2, 1.1 Hz, 2H), 6.62 (ddd, *J* = 7.3, 7.3, 1.2 Hz, 2H), 6.15 (dd, *J* = 7.4, 0.8 Hz, 2H), 6.04 (dd, *J* = 7.6, 0.5 Hz, 2H), 2.48–2.28 (m, 36H), 1.90–1.79 (m, 24H); ^13^C-NMR (75 MHz, CDCl_3_): δ ppm 157.0, 156.8, 145.9, 143.9, 136.3, 135.8, 132.9, 132.3, 127.24, 127.22, 121.08, 121.06, 120.8, 120.6, 113.9, 113.7, 61.9, 61.4, 42.9, 42.8, 35.79, 35.75, 29.49, 29.46; ESI-MS (ES+, CHCl_3_/CH_3_OH): *m/z* = 807.0 [(M/2)+Na]^+^

*[(**1**)(**2**)Ir-µ-Cl]_2_*: A mixture 3/1 of 2-methoxyethanol/water (12 mL) was deoxygenated by bubbling nitrogen through it for 10-15 min. The ligands **1** (40 mg, 0.25 mmol) and **2** (70.2 mg, 0.25 mmol), and IrCl_3_⊕3H_2_O (62.5 mg, 0.21 mmol) were added and the solution was heated to 97 ºC in the dark for 24 hours, under nitrogen atmosphere (without bubbling). After cooling to room temperature, the yellow solid was filtered off, washed with water (3 × 80 mL) and dried. This reaction is expected to yield a mixture of chloride-bridged complexes [(**X**)(**Y**)Ir-µ-Cl]_2_ (**X**=**Y**=**1**,**2**). The reaction was followed by TLC (eluent: CH_3_OH/CHCl_3_ 20/80) where several spots, corresponding to different dimeric species, could be observed. The crude material was further reacted without purification.

*[Ir(**1**)_2_(pytl-Me)]Cl*: To a suspension of the precursor [(**1**)_2_Ir-µ-Cl]_2_ (47.2 mg, 0.043 mmol) in MeOH/CHCl_3_ 1/3 v/v (4 mL) was added pytl-Me as a solid (14.6 mg, 0.086 mmol). The suspension was heated to 45 ºC and stirred in the dark for 3 hours, after which time a clear and yellow solution was obtained. The reaction was followed by TLC (eluent: MeOH/CHCl_3_ 30/70) where, under UV light at 366 nm, the compound appeared as a bright green-blue luminescent spot. After removal of the solvent *in vacuo*, the solid obtained was purified by PLC (eluent: MeOH/CHCl_3_ 25/75). The product was obtained as a slightly yellow solid (47.4 mg, 80%). ^1^H-NMR (400 MHz, CDCl_3_): δ ppm 10.48 (s, 1H), 8.88 (d, *J* = 7.9 Hz, 1H), 7.93-7.87 (m, 2H), 7.77 (s, 1H), 7.73 (s, 1H), 7.43 (dd, *J* = 7.5, 1.1 Hz, 1H), 7.39 (dd, *J* = 7.4, 1.1 Hz, 1H), 7.12 (ddd, *J* = 7.5, 5.6, 1.3 Hz, 1H), 6.96 (ddd, *J* = 7.5, 7.5, 1.2 Hz, 1H), 6.92 (ddd, *J* = 7.4, 7.4, 1.2 Hz, 1H), 6.85 (ddd, *J* = 7.5, 7.5, 1.4 Hz, 1H), 6.81 (ddd, *J* = 7.5, 7.5, 1.4 Hz, 1H), 6.32–6.28 (m, 2H), 4.16 (s, 3H), 4.08 (s, 3H), 4.05 (s, 3H); ^13^C-NMR (75 MHz, CDCl_3_): δ ppm 157.9, 157.5, 151.1, 150.3, 149.3, 146.1, 142.3, 139.0, 135.3, 135.0, 132.75, 132.73, 128.9, 128.6, 128.1, 124.8, 123.7, 122.6, 122.3, 122.2, 122.1, 118.9, 118.8, 38.60, 38.56, 38.5; HRMS (ES+, CH_3_OH): *m/z* calcd for C_26_H_24_IrN_10_: 669.18146; found: 669.18464 M^+^.

*[Ir(**2**)_2_(pytl-ada)]Cl*: To a suspension of the precursor [(**2**)_2_Ir-µ-Cl]_2_ (20.0 mg, 0.013 mmol) in MeOH/CHCl_3_ 1/3 v/v (4 mL) was added pytl-ada as a solid (7.29 mg, 0.026 mmol). The suspension was heated to 45 ºC and stirred in the dark for 3 hours, after which time a clear and yellow solution was obtained. The reaction was followed by TLC (eluent: MeOH/CHCl_3_ 20/80) where, under UV light at 366 nm, the compound appeared as a bright green-blue luminescent spot. After removal of the solvent *in vacuo*, the solid obtained was purified by column chromatography (eluent: from CHCl_3_ to MeOH/CHCl_3_ 5/95). The product was obtained as a slightly yellow solid (20.3 mg, 76%). ^1^H-NMR (400 MHz, CDCl_3_): δ ppm 10.56 (s, 1H), 9.16 (d, *J* = 7.8 Hz, 1H), 7.96 (bd, *J* = 5.4 Hz, 1H), 7.92 (ddd, *J* = 7.8, 7.8, 1.4 Hz, 1H), 7.76 (s, 1H), 7.71 (s, 1H), 7.34 (dd, *J* = 5.7, 1.3 Hz, 1H), 7.32 (dd, *J* = 5.6, 1.2 Hz, 1H), 7.07 (ddd, *J* = 7.2, 5.8, 1.2 Hz, 1H), 6.91 (ddd, *J* = 6.7, 6.7, 1.2 Hz, 1H), 6.88 (ddd, *J* = 6.6, 6.6, 1.2 Hz, 1H), 6.82 (ddd, *J* = 7.4, 7.4, 1.4 Hz, 1H), 6.77 (ddd, *J* = 7.5, 7.5, 1.4 Hz, 1H), 6.16 (dd, *J* = 7.6, 0.6 Hz, 1H), 6.13 (dd, *J* = 7.5, 0.6 Hz, 1H), 2.30–2.19 (m, 15H), 2.13–2.10 (m, 12H), 1.81–1.68 (m, 18H); ^13^C-NMR (75 MHz, CDCl_3_): δ ppm 156.7, 156.1, 151.7, 150.0, 148.9, 146.8, 143.3, 138.9, 136.1, 135.7, 133.0, 132.3, 128.2, 127.4, 125.2, 124.01, 123.95, 122.1, 121.7, 121.5, 121.2, 114.1, 114.0, 62.2, 61.9, 61.8, 42.70, 42.67, 42.5, 35.67, 35.63, 29.7, 29.5, 29.37, 29.36; HRMS (ES+, CH_3_OH): *m/z* calcd for C_53_H_60_IrN_10_: 1029.46316; found: 1029.46242 M^+^.

**Scheme 7:**
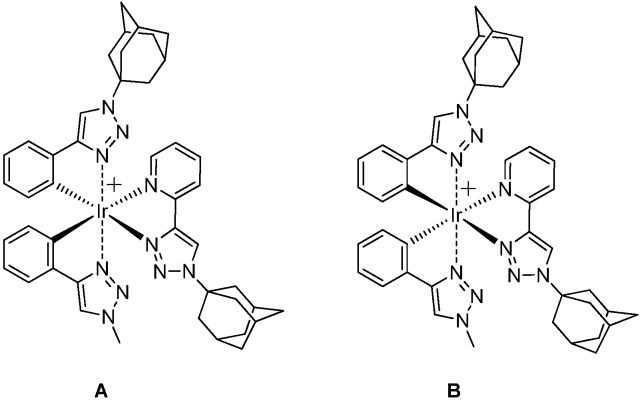
Structure of the diastereoisomers **A** and **B** of [Ir(**1**)(**2**)(pytl-ada)]Cl.

*[Ir(**1**)(**2**)(pytl-ada)]Cl*: To a suspension of the crude mixture of the precursors [(**X**)(**Y**)Ir-µ-Cl]_2_ (**X**=**1**,**2**; **Y=1**,**2**) (76.2 mg) in MeOH/CHCl_3_ 1/3 v/v (8 mL) was added pytl-ada as a solid (44.8 mg, 0.16 mmol). The suspension was heated to 45 ºC and stirred in the dark for 3 hours, after which time a yellow solution was obtained. The reaction was followed by TLC (eluent: MeOH/CHCl_3_ 20/80). The mixture of products obtained could not be separated by column chromatography. After removal of the solvent *in vacuo*, the crude material was purified by using a semipreparative HPLC equipped with a reversed-phase column. The mobile phase was a gradient of H_2_O and acetonitrile (50% for 20 min + gradient 50% to 100% v/v of acetonitrile in 40 min) both containing 0.1% v/v HCl. The complex [Ir(**1**)(**2**)(pytl-ada)]Cl exists in four stereoisomeric forms: the two diastereoisomers **A** and **B** (Scheme 7), and their corresponding enantiomers (Λ,Δ). The diastereoisomeric species **A**(Λ+Δ) and **B**(Λ+Δ) could be separated by HPLC in the conditions described above. However, we did not perform their isolation and collected them as a mixture. The HPLC fractions containing the desired iridium complex were identified by ESI-MS. The retention time of the two peaks of [Ir(**1**)(**2**)(pytl-ada)]Cl were t = 41.9 min and t = 43.3 min. After collecting the fractions, the solvent was removed *in vacuo* at 40 ºC. The product was obtained as a slightly yellow solid (21.1 mg, overall yield = 11%) containing the two diastereoisomers in a ratio 1:0.8 as calculated from the NMR spectrum. ^1^H-NMR (400 MHz, CDCl_3_): δ ppm 10.49 (s, 0.8H), 10.45 (s, 1H), 9.12–9.05 (m, 1H+0.8H), 7.93–7.83 (m, 2H+1.6H), 7.77 (bs, 1.6H), 7.76 (s, 1H), 7.73 (s, 1H), 7.33 (dd, *J* = 7.4, *J* = 1.1 Hz, 1.6H), 7.32 (dd, *J* = 7.2, *J* = 1.1 Hz, 1H), 7.29 (dd, *J* = 7.4, *J* = 1.2 Hz, 1H), 7.08–7.02 (m, 1H+0.8H), 6.92–6.76 (m, 3H+2.4H), 6.75 (ddd, *J* = 7.4, *J* = 7.4, *J* = 1.3 Hz, 0.8H) 6.74 (ddd, *J* = 7.4, *J* = 7.4, *J* = 1.3 Hz, 1H), 6.31 (d, *J* = 7.5 Hz, 0.8H), 6.24 (d, *J* = 7.5 Hz, 1H), 6.12 (d, *J* = 7.5 Hz, 1H), 6.08 (d, *J* = 7.5 Hz, 0.8H), 4.02 (s, 2.4H), 4.00 (s, 3H), 2.28–2.22 (m, 9H+7.2H), 2.22–2.17 (m, 3H+2.4H), 2.14–2.09 (m, 6H+4.8H), 1.82–1.66 (m, 12H+9.6H); ^13^C-NMR (75 MHz, CDCl_3_): δ ppm 157.8, 157.2, 156.6, 156.0, 151.48, 151.47, 150.1, 149.9, 148.84, 148.79, 146.5, 146.2, 143.2, 142.9, 138.9–138.8 (m), 135.9, 135.63, 135.60, 135.4, 133.1, 132.9, 132.4, 132.2, 128.2, 128.1, 127.5, 127.3, 125.1, 124.9, 124.3, 124.2, 123.7, 123.6, 122.23, 122.19, 122.1, 121.8, 121.7, 121.56, 121.55, 121.3, 119.2, 119.0, 114.3, 114.1, 62.4, 62.3, 61.9, 61.8, 42.62, 42.57, 42.4, 42.3, 38.5, 38.4, 35.60, 35.58, 35.55, 29.41–29.37 (m), 29.32, 29.30; HRMS (ES+, CH_3_OH): *m/z* calcd for C_44_H_48_IrN_10_: 909.36926; found: 909.37012 M^+^.

*[Ir(**1**)_2_(pytl-DC)]Cl*: To a suspension of the precursor [(**1**)_2_Ir-µ-Cl]_2_ (28.3 mg, 0.026 mmol) in MeOH/CHCl_3_ 1/3 v/v (4 mL) was added pytl-DC as a solid (26.8 mg, 0.052 mmol). The suspension was heated to 45 ºC and stirred in the dark for 17 hours, after which time a clear and yellow solution was obtained. The reaction was followed by TLC (eluent: MeOH/CHCl_3_ 20/80) where, under UV light at 366 nm, the compound appeared as a bright green-blue luminescent spot. After removal of the solvent *in vacuo*, the solid obtained was purified by PLC (eluent: MeOH/CHCl_3_ 20/80). The product was obtained as a slightly yellow solid (39.1 mg, 73%) containing a mixture 1:1 of two diastereoisomers as also confirmed by the NMR spectrum.^ 1^H-NMR (400 MHz, CD_3_OD): δ ppm 9.041 (s, 1H), 9.038 (s, 1H), 8.24–8.23 (m, 1H), 8.23 –8.22 (m, 3H), 8.20–8.16 (m, 2H), 8.08–7.99 (m, 4H), 7.50–7.46 (m, 2H), 7.44–7.40 (m, 2H), 7.34–7.30 (m, 2H), 6.97–6.92 (m, 2H), 6.88–6.79 (m, 4H), 6.71 (ddd, *J* = 7.4, *J* = 1.4, *J* = 1.4 Hz, 2H), 6.29 (ddd, *J* = 7.5, *J* = 1.1, *J* = 0.5 Hz, 1H), 6.28 (ddd, *J* = 7.6, *J* = 1.1, *J* = 0.5 Hz, 1H), 6.25–6.22 (m, 2H), 4.81–4.76 (m, 2H), 4.07–4.06 (m, 6H), 4.05 (bs, 6H), 3.51–3.42 (m, 2H), 2.37–0.89 (m, 64H), 0.72 (s, 6H); HRMS (ES+, CH_3_OH): *m/z* calcd for C_49_H_60_IrN_10_O_3_: 1029.44791; found: 1029.44633 M^+^.

*[Ir(**1**)_2_(pytl-βCD)]Cl*: To a suspension of the precursor [(**1**)_2_Ir-µ-Cl]_2_ (7.04 mg, 0.006 mmol) in MeOH/CHCl_3_ 1/3 v/v (4 mL) was added pytl-βCD as a solid (20.1 mg, 0.013 mmol). The suspension was heated to 45 ºC and stirred in the dark for 3 hours, after which time a clear and yellow solution was obtained. The reaction was followed by TLC (eluent: MeOH/CHCl_3_ 15/85) where, under UV light at 366 nm, the compound appeared as a bright green-blue luminescent spot. After removal of the solvent *in vacuo*, the solid obtained was purified by column chromatography (eluent: MeOH/CHCl_3_ 5/95). The product was obtained as a slightly yellow solid (16.0 mg, 59%) containing a mixture 1:1 of two diastereoisomers as also confirmed by the NMR spectrum. ^1^H-NMR (300 MHz, CDCl_3_): δ ppm 9.73 (s, 1H), 9.63 (s, 1H), 8.87–8.68 (m, 2H), 7.95–7.87 (m, 2H), 7.81 (s, 1H), 7.78 (s, 1H), 7.744 (s, 1H), 7.736 (s, 1H), 7.40–7.21 (m, 6H), 7.17–7.11 (m, 2H), 6.97–6.65 (m, 8H), 6.30–6.21 (m, 2H), 6.17–6.06 (m, 2H), 5.57–5.46 (m, 1H), 5.39–5.03 (m, 13H), 4.95–4.83 (m, 1H), 4.78–4.69 (m, 1H), 4.17 (s, 3H), 4.12 (s, 3H), 4.11 (s, 3H), 4.05 (s, 3H), 4.15–2.98 (m, 200H), 2.96–2.89 (m, 1H), 2.81–2.74 (m, 1H); HRMS (ES+, CH_3_OH): *m/z* calcd for C_87_H_130_IrN_10_O_34_: 2051.83801; found: 2051.83965 M^+^.

## Supplementary Materials

Supplementary File 1
