# Supplementary material for: Cationic Heteroleptic Cyclometalated IridiumIII Complexes Containing Phenyl-Triazole and Triazole-Pyridine Clicked Ligands
Source: Molecules. 2010 Mar 23;15(3):2039–59. doi: 10.3390/molecules15032039 (PMC6257372; doi:10.3390/molecules15032039)
Supplement: Supplementary File 1 [file molecules-15-02039-s001.pdf]

Supporting information

Cationic Heteroleptic Cyclometalated Iridium^{III} Complexes Containing Phenyl-Triazole and Triazole-Pyridine Clicked Ligands

Marco Felici [1], Pablo Contreras-Carballada [2], Jan M. M. Smits [3], Roeland J. M. Nolte [1], René M. Williams [2], Luisa De Cola [4] and Martin C. Feiters [1,*]

Table of Contents

X-ray crystallographic analysis and results 2

UV-Vis and Emission spectra 39

X-ray crystallographic analysis and results

X-ray crystallography: Single crystals of **1** (phtl-Me) and **2** (phtl-ada) were grown by slow evaporation of a solution of compounds **1** and **2** in $CHCl_3$/heptane. Single crystals of pytl-DC were grown by slow evaporation of a solution of pytl-DC in CH_3OH. Single crystals of **6a** and **7a** were grown by slow evaporation of a slightly acidic solution of **6a** and **7a** in H_2O/Acetonitrile. The crystal data and summaries of the data collection and structure refinement are given in Table S1 for compounds **1** and **2**, and Table S2 for **6a**, **7a**, and pytl-DC; selected distances and bond angles as well as atomic coordinates and equivalent isotropic displacement parameters for the non-hydrogen atoms are also given. All measurements were performed at -65°C. The structures of **1**, **6a**, and **7a** were solved by the program SHELXS [1], those of **2** and pytl-DC by the program CRUNCH [2]. All non-hydrogen atoms were refined with anisotropic temperature factors. The hydrogen atoms were placed at calculated positions, and refined isotropically in riding mode.

For compound **1** (phtl-Me), PLATON [3] and CheckCIF suggest that the space group should be Pcab instead of $Pc2_1b$. However, in the diffraction pattern reflections h0l, h=2n+1 are clearly present, although they are very weak. Refinement in space group Pcab resulted in a final R value of 0.0923 as compared to 0.0606 in space group . There is a small difference in the torsion angles around the C4-C7 bond in the two independent molecules in space group $Pc2_1b$, 26.14 and 21.64 degrees for molecules A and B respectively. Constrained positions giving the best possible match with electron densities in the difference Fourier map for the hydrogen atoms on atoms C6A and C6B show a marked deviation from glide plane symmetry although it should be pointed out that the difference Fourier map does not show unambiguous positions. Based on these considerations we decided that the space group should be $Pc2_1b$ with pseudo Pcab symmetry.

Interestingly there is a large difference in the dihedral angles between the least square planes through the triazole and phenyl rings in **1** (Figure S1, Table S3) and **2** (Figure S2, Table S4). The value of 6.13 deg. for **2** is in the same range as that reported by us [4] for the analogous pytl (pyridine-triazole) compounds (methyl derivative 7.56-9.27 deg., adamantane 2.90 deg.) whereas the values for the two molecules of **1** are 23.52 and 24.00 deg. The shortest bond in the triazole rings is that between the two nitrogens that are only connected to other ring atoms, which qualifies the electronic structure of these rings as 'azo-like' [5].

For compound **7a**, the hydrogen atoms were placed at calculated positions, and refined isotropically in riding mode, as for the other compounds, except H2A which was refined freely.

For compound **6a**, it was pretty difficult to find a crystal suitable for data collection. The structure shows disorder, especially in the side chains, resulting in rather large anisotropic thermal displacement parameters. Attempts to parameterize this disorder resulted in an unstable refinement. It was not possible to find hydrogen atoms for O1 and O2 in the difference Fourier map, and the constrained positions of H1 and H2A are not very reliable. Although it is likely for O1-H1 to form a hydrogen bond, no such bond could be found with the present parameters for H1. Likewise the hydrogen bond reported for O2-H2A should be treated with caution. The structure showed two voids of 120 $Å^3$, each containing 27 electrons. The SQUEEZE procedure from PLATON [3] was used to account for these electron densities. No possible solvent was proposed for these electron densities and therefore it was not possible to account for them in the physical constants for this structure. Table S6e contains possible but somewhat questionable hydrogen bonds. It should be noted that the value of the Flack parameter 0.0(8) (in particular the large uncertainty in it) does not allow a decision between the possible enantiomers to be made. This does not affect our conclusion that the azide is in an axial position; combined with the knowledge of the configuration of all the other non-affected chiral centers in the starting material this allows us to choose the enantiomer represented in Figure 1 in the manuscript as the structure of compound **6a**.

As the hydroxyl groups of all deoxycholic acid isomers are known to be involved in intramolecular hydrogen bonds [6], and **6a** (Figure S5, Table S6) has the configuration of isoursodeoxycholic acid while **7a** (Figure S4, Table S5) and pytl-DC (Figure S5, Table S7) have that of chenodeoxycholic acid [7], it is of interest to look at the effect of the substitution of the hydroxy groups in these compounds on the intermolecular hydrogen bonding. The azide moiety in **6a** and **7a** does not participate in H-bonds; **7a** is found to give dimers where the –OH of one molecule is H-bonded to the carboxylic acid moiety of the other, and *vice versa*. As mentioned above, the H-bonding pattern for **6a** could not be established unambiguously. For pytl-DC, the hydroxyl group is not involved in H-bonding, whereas the pyridine triazole moiety, which has a transoid conformation, can accept hydrogen bonds either at the pyridine or one of the triazole nitrogens. As a result there is a large spread in the dihedral angles beween the least square planes through the pyridine and triazole rings for the three molecules; the values are 11.07, 15.56, and 29.68 deg. For all molecules, the shortest bond in the triazole rings for all

molecules is that between the nitrogen atoms that are only bound to other ring atoms, which qualifies the electronic structure as 'azo-like'.

For compound pytl-DC, no possible hydrogen bond was found for O1C-H1C. This hydrogen atom was placed at a constrained position giving the best possible match with electron density in the difference Fourier map. This map showed no useful alternative maximum near O1C. Moreover, no suitable acceptor is located within 3.6 Å from O1C. We therefore accept the position of H1C as given here.

For all compounds, geometrical calculations [3] revealed neither unusual geometric features, nor unusual short intermolecular contacts. The calculations revealed no higher symmetry and no (further) solvent accessible areas.

Crystallographic data (excluding structure factors) for the structures reported in this paper have been deposited with the Cambridge Crystallographic Data Centre as supplementary publication CCDC-1003/, deposition codes: **1** (FELIC3), 757888; **2** (MFR130), 757886; **6a** (MFR99), 757884; **7a** (MFR99B), 757885; pytl-DC (PYTLUD), 757887. Copies of available material can be obtained, free of charge, on application to the Director, CCDC, 12 Union Road, Cambridge CB2 1EZ, UK, (fax: +44-(0) 1223-336033 or e-mail:teched@chemcrys.cam.ac.uk).

Figure S1. Structure and atomic numbering of phtl-Me (**1**) produced with PLATON [3].

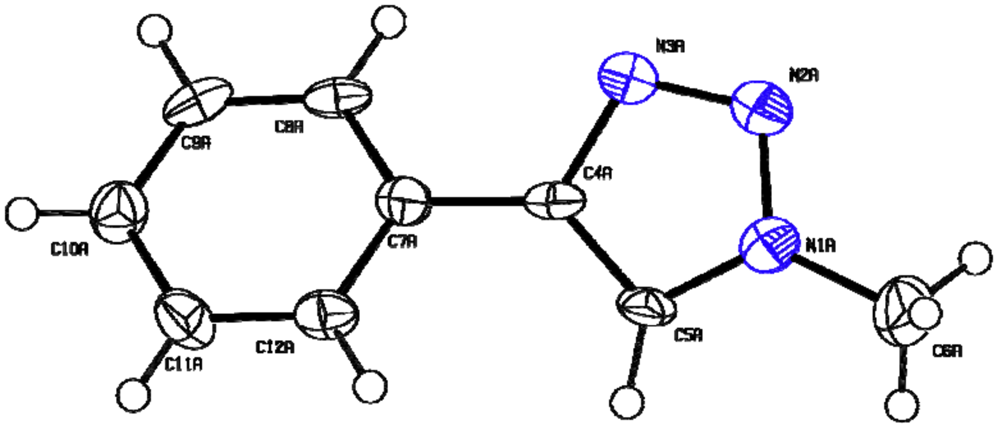

Figure S2. Structure and atomic numbering of phtl-ada (**2**) produced with PLATON [3].

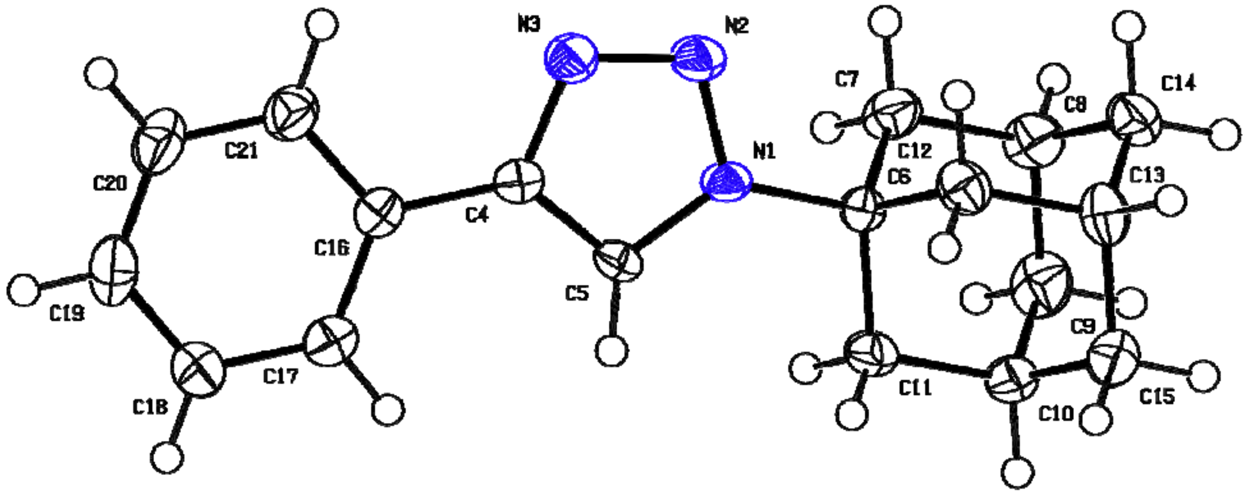

Figure S3. Structure and atomic numbering of **6a** produced with PLATON [3]

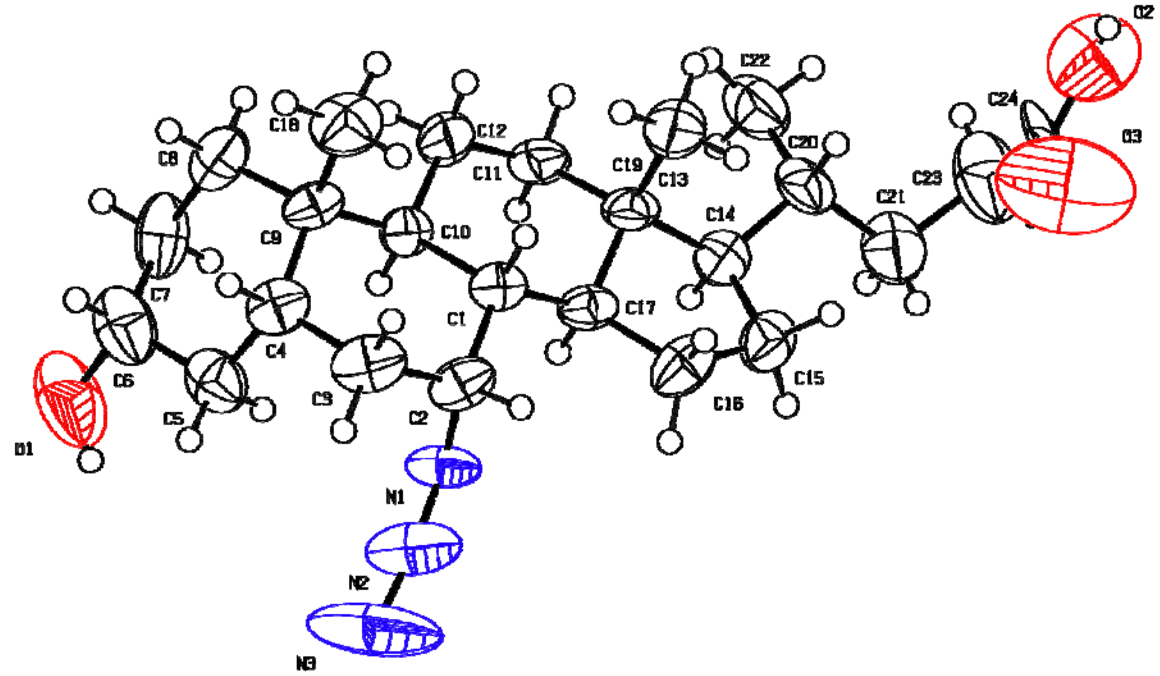

Figure S4. Structure and atomic numbering of **7a** produced with PLATON [3]

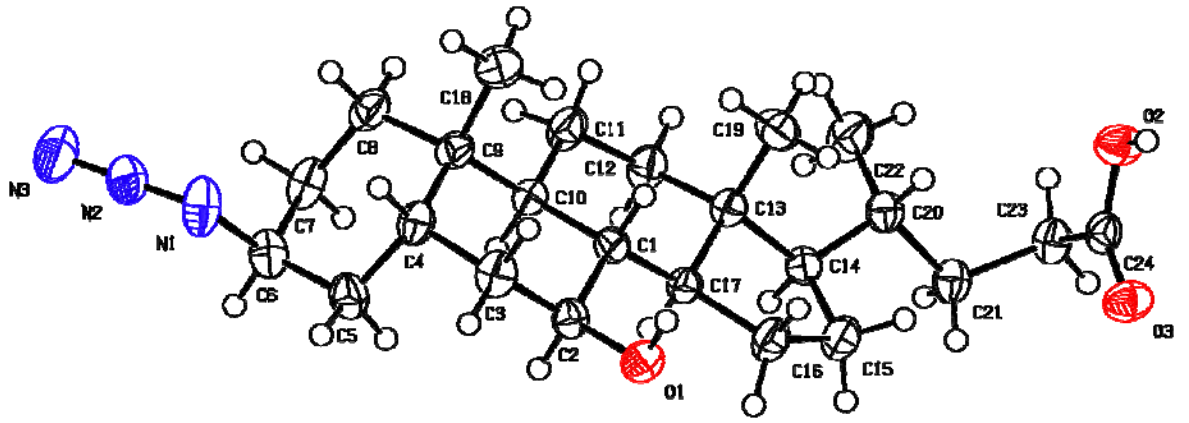

Figure S5. Structure and atomic numbering of pytl-DC produced with PLATON [3]

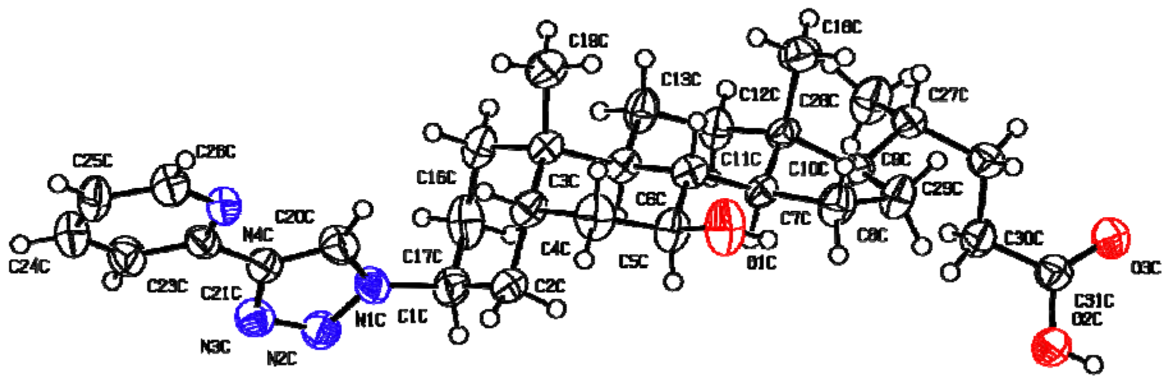

Table S1. Data for the crystallographic structure determination of **1** and **2**.

	**1** (phtl-Me)	**2** (phtl-ada)
Identification code	FELIC3	MFR130
Crystal colour	translucent colourless	translucent colourless
Crystal shape	rather regular rod	rather regular needle
Crystal size	0.22 x 0.10 x 0.05 mm	0.25 x 0.07 x 0.04 mm
Empirical formula	$C_9H_9N_3$	$C_{18}H_{21}N_3$
Formula weight	159.19	279.38

Temperature	208(2) K	208(2) K
Radiation / Wavelength	Mo*Kα* (graphite mon.) / 0.71073 Å	Mo*Kα* (graphite mon.) / 0.71073 Å
Crystal system, space group	Orthorhombic, $Pc2_1b$	Monoclinic, $P2_1/a$
Unit cell dimensions	a = 5.6729(2) Å<br>b = 14.2177(8) Å<br>c = 20.0007(17) Å	a = 11.0480(4) Å<br>b = 11.8690(6) Å<br>β = 92.896(4) deg<br>c = 11.2655(7) Å
Volume	1613.17(17) $Å^3$	1475.34(13) $Å^3$
Z, Calculated density	8, 1.311 Mg/m^3	4, 1.258 Mg/m^3
Absorption coefficient	0.083 mm^{-1}	0.076 mm^{-1}
Diffractometer / scan	Nonius KappaCCD with area detector ϕ and ω scan	Nonius KappaCCD with area detector ϕ and ω scan
F(000)	672	600
θ range for data collection	2.49 to 27.51 deg.	2.49 to 25.00 deg.
Index ranges	7<=h<=7, -18<=k<=17, -25<=l<=19	-13<=h<=12, -14<=k<=13, -13<=l<=13
Reflections collected / unique	17219 / 3501 [$R_{(int)}$ = 0.0447]	18071 / 2597 [$R_{(int)}$ = 0.0790]
Reflections observed	2314 ([$I_o>2\sigma(I_o)$])	1719 ([$I_o>2\sigma(I_o)$])
Completeness to 2θ = 25.00	99.7%	94.6%
Absorption correction	SADABS multiscan correction [8]	SADABS multiscan correction [8]
Refinement method	Full-matrix least-squares on F^2	Full-matrix least-squares on F^2
Computing	SHELXL-97 [9]	SHELXL-97 [9]
Data / restraints / parameters	3501 / 1 / 219	2597 / 0 / 190
Goodness-of-fit on F^2	1.091	1.146
SHELXL-97 weight parameters	0.0394, 0.3062	0.0399, 0.7649
Final R indices [$I>2\sigma(I)$]	R_1 = 0.0606, wR_2 = 0.0983	R_1 = 0.0706, wR_2 = 0.1125
R indices (all data)	R_1 = 0.1050, wR_2 = 0.1108	R_1 = 0.1240, wR_2 = 0.1296
Largest diff. peak and hole	0.158 and -0.223 e.$Å^{-3}$	0.233 and -0.297 e. $Å^{-3}$

Table S2. Data for the crystallographic structure determination of **6a**, **7a** and pytl-DC.

	**7a**	**6a**	pytl-DC
Identification code	MFR99B	MFR99	PYTLUD
Crystal colour	translucent colourless	translucent colourless	translucent colourless
Crystal shape	rather regular rod	rough fragment	rather regular fragment

Crystal size	0.28 x 0.14 x 0.07 mm	0.19 x 0.18 x 0.08 mm	0.29 x 0.25 x 0.16 mm
Empirical formula	$C_{24}H_{39}N_3O_3$	$C_{24}H_{39}N_3O_3$	$C_{31}H_{44}N_4O_3$
Formula weight	417.58	417.58	520.70
Temperature	208(2) K	208(2) K	208(2) K
Radiation / Wavelength	Cu$K\alpha$ (graphite mon.) / 1.54184 Å	Cu$K\alpha$ (graphite mon.) / 1.54184 Å	Mo$K\alpha$ (graphite mon.) / 0.71073 Å
Crystal system, space group	Monoclinic, $P2_1$	Monoclinic, C2	Orthorhombic, $P2_12_12_1$
Unit cell dimensions	a = 11.8912(3) Å<br>b = 6.2752(2) Å<br>β = 97.900(2) deg<br>c = 15.3649(3) Å	a = 20.7894(4) Å<br>b = 7.8350(2) Å<br>β = 105.447(3) deg<br>c = 15.5366(3) Å	a = 11.1262(14) Å<br>b = 11.5765(14) Å<br>c = 64.786(7) Å
Volume	1135.64(5) $Å^3$	2439.26(9) $Å^3$	8344.6(17) $Å^3$
Z, Calculated density	2, 1.221 Mg/m^3	4, 1.137 Mg/m^3	12, 1.243 Mg/m^3
Absorption coefficient	0.636 mm^{-1}	0.593 mm^{-1}	0.081 mm^{-1}
Diffractometer / scan	Nonius KappaCCD with area detector ϕ and ω scan	Nonius KappaCCD with area detector ϕ and ω scan	Nonius KappaCCD with area detector ϕ and ω scan
F(000)	456	912	3384
θ range for data collection	2.90 to 64.99 deg.	2.95 to 54.23 deg.	2.06 to 27.51 deg.
Index ranges	-13<=h<=13, -7<=k<=6, -18<=l<=17	-21<=h<=20, -8<=k<=8, -16<=l<=16	-14<=h<=14, -14<=k<=13, -82<=l<=83
Reflections collected / unique	9170 / 3557 [$R_{(int)}$ = 0.0249]	7626 / 2785 [$R_{(int)}$ = 0.0341]	40445 / 14003 [$R_{(int)}$ = 0.0385]
Reflections observed	3303 ([$I_o>2\sigma(I_o)$])	2310 ([$I_o>2\sigma(I_o)$])	8397 ([$I_o>2\sigma(I_o)$])
Completeness	to 2theta = 64.99 99.1%	to 2θ = 50.00 49.7%	to 2θ = 25.00 90.5%
Absorption correction	SADABS multiscan correction [8]	SADABS multiscan correction [8]	SADABS multiscan correction [8]
Refinement method	Full-matrix least-squares on F^2	Full-matrix least-squares on F^2	Full-matrix least-squares on F^2
Computing	SHELXL-97 [9]	SHELXL-97 [9]	SHELXL-97 [9]
Data / restraints / parameters	3557 / 1 / 278	2785 / 1 / 277	14003 / 0 / 1042
Goodness-of-fit on F^2	1.080	1.050	1.038
SHELXL-97 weight parameters	0.0384, 0.1567	0.0973, 2.1280	0.0379, 1.1082
Final R indices	R_1 = 0.0321, wR_2 =	R_1 = 0.0620, wR_2 =	R_1 = 0.0504, wR_2 =

[I>2σ(I)]	0.0778	0.1647	0.0860
R indices (all data)	R_1 = 0.0359, wR_2 = 0.0804	R_1 = 0.0746, wR_2 = 0.1753	R_1 = 0.1139, wR_2 = 0.1024
Largest diff. peak and hole	0.121 and -0.174 e. $Å^{-3}$	0.432 and -0.184 e. $Å^{-3}$	0.278 and -0.186 e. $Å^{-3}$

Table S3. Crystallographic data for phtl-Me (**1**) (FELIC3)

Table S3a. Atomic coordinates (x 10^4) and equivalent isotropic displacement parameters ($Å^2$ x 10^3) for FELIC3. U(eq) is defined as one third of the trace of the orthogonalized U_{ij} tensor.

	x	y	z	U(eq)
N(1A)	3148(6)	6190(3)	5410(1)	37(1)
N(2A)	829(6)	6091(3)	5341(1)	41(1)
N(3A)	-96(6)	6137(3)	5949(1)	38(1)
C(4A)	1707(6)	6177(3)	6404(2)	25(1)
C(5A)	3776(6)	6211(3)	6052(2)	30(1)
C(6A)	4681(7)	6162(4)	4824(2)	50(1)
C(7A)	1234(6)	6201(3)	7128(1)	28(1)
C(8A)	-763(6)	5832(3)	7400(2)	26(1)
C(9A)	-1137(7)	5848(3)	8074(2)	31(1)
C(10A)	494(6)	6259(3)	8502(2)	34(1)
C(11A)	2548(8)	6627(3)	8231(2)	36(1)
C(12A)	2936(6)	6615(3)	7551(2)	29(1)
N(1B)	1793(6)	8750(3)	4670(1)	36(1)
N(2B)	4169(6)	8776(4)	4745(1)	46(1)
N(3B)	5054(6)	8801(3)	4141(1)	37(1)
C(4B)	3307(7)	8699(4)	3693(2)	29(1)
C(5B)	1230(7)	8667(4)	4026(2)	37(1)
C(6B)	296(7)	8726(5)	5257(2)	56(2)
C(7B)	3689(6)	8665(3)	2965(1)	21(1)
C(8B)	5770(7)	9050(4)	2690(2)	35(1)
C(9B)	6147(8)	9027(4)	2009(2)	40(1)
C(10B)	4491(7)	8637(4)	1585(2)	37(1)
C(11B)	2480(7)	8246(3)	1858(2)	31(1)
C(12B)	2107(6)	8283(3)	2532(2)	30(1)

Table S3b. Selected bond lengths [Å] and angles [deg] for FELIC3.

N(1A)-N(2A)	1.330(4)
N(1A)-C(5A)	1.334(4)
N(1A)-C(6A)	1.460(4)
N(2A)-N(3A)	1.325(4)
N(3A)-C(4A)	1.371(4)
C(4A)-C(5A)	1.369(5)
C(4A)-C(7A)	1.472(4)
C(7A)-C(8A)	1.362(5)
C(7A)-C(12A)	1.413(5)
C(8A)-C(9A)	1.365(5)
C(9A)-C(10A)	1.389(5)
C(10A)-C(11A)	1.386(5)

C(11A)-C(12A)	1.378(4)
N(1B)-C(5B)	1.331(4)
N(1B)-N(2B)	1.357(4)
N(1B)-C(6B)	1.450(4)
N(2B)-N(3B)	1.309(4)
N(3B)-C(4B)	1.344(5)
C(4B)-C(5B)	1.355(5)
C(4B)-C(7B)	1.472(4)
C(7B)-C(12B)	1.360(5)
C(7B)-C(8B)	1.413(5)
C(8B)-C(9B)	1.380(5)
C(9B)-C(10B)	1.381(6)
C(10B)-C(11B)	1.382(5)
C(11B)-C(12B)	1.366(5)
N(2A)-N(1A)-C(5A)	111.5(3)
N(2A)-N(1A)-C(6A)	120.2(3)
C(5A)-N(1A)-C(6A)	128.0(3)
N(3A)-N(2A)-N(1A)	107.0(3)
N(2A)-N(3A)-C(4A)	108.4(3)
C(5A)-C(4A)-N(3A)	107.5(3)
C(5A)-C(4A)-C(7A)	131.4(3)
N(3A)-C(4A)-C(7A)	121.2(3)
N(1A)-C(5A)-C(4A)	105.4(3)
C(8A)-C(7A)-C(12A)	119.3(3)
C(8A)-C(7A)-C(4A)	122.4(3)
C(12A)-C(7A)-C(4A)	118.3(3)
C(7A)-C(8A)-C(9A)	121.2(3)
C(8A)-C(9A)-C(10A)	120.7(3)
C(11A)-C(10A)-C(9A)	118.6(3)
C(12A)-C(11A)-C(10A)	120.9(4)
C(11A)-C(12A)-C(7A)	119.2(3)
C(5B)-N(1B)-N(2B)	110.4(3)
C(5B)-N(1B)-C(6B)	129.8(4)
N(2B)-N(1B)-C(6B)	119.5(3)
N(3B)-N(2B)-N(1B)	106.2(3)
N(2B)-N(3B)-C(4B)	109.3(3)
N(3B)-C(4B)-C(5B)	108.5(3)
N(3B)-C(4B)-C(7B)	123.6(3)
C(5B)-C(4B)-C(7B)	127.9(3)
N(1B)-C(5B)-C(4B)	105.3(3)
C(12B)-C(7B)-C(8B)	117.3(3)
C(12B)-C(7B)-C(4B)	123.0(4)
C(8B)-C(7B)-C(4B)	119.7(3)
C(9B)-C(8B)-C(7B)	120.3(4)
C(8B)-C(9B)-C(10B)	120.7(4)
C(9B)-C(10B)-C(11B)	118.8(3)
C(12B)-C(11B)-C(10B)	120.1(4)
C(7B)-C(12B)-C(11B)	122.8(4)

Table S3c. Hydrogen coordinates (x 10^4) and isotropic displacement parameters (Å^2 x 10^3) for FELIC3.

	x	y	z	U(eq)
H(5A)	5313	6242	6227	36
H(6A1)	3970	5768	4483	75
H(6A2)	6202	5905	4949	75
H(6A3)	4888	6794	4651	75
H(8A)	-1904	5561	7119	31
H(9A)	-2515	5577	8251	37

H(10A)	212	6288	8964	41
H(11A)	3693	6887	8516	43
H(12A)	4315	6879	7371	35
H(5B)	-286	8601	3843	44
H(6B1)	-1230	8468	5138	84
H(6B2)	95	9360	5428	84
H(6B3)	1022	8336	5597	84
H(8B)	6902	9324	2973	42
H(9B)	7546	9278	1831	48
H(10B)	4728	8638	1120	44
H(11B)	1365	7955	1579	37
H(12B)	698	8034	2705	36

Table S3d. Anisotropic displacement parameters ($Å^2$ x 10^3) for FELIC3. The anisotropic displacement factor exponent takes the form: $-2 \pi^2 [h^2 a^{*2} U_{11} + ... + 2 h k a^* b^* U_{12}]$

	U_{11}	U_{22}	U_{33}	U_{23}	U_{13}	U_{12}
N(1A)	28(2)	49(2)	33(2)	2(2)	3(1)	-5(2)
N(2A)	30(2)	60(3)	32(2)	2(2)	-5(1)	2(2)
N(3A)	26(2)	56(3)	32(1)	-6(2)	-4(1)	0(2)
C(4A)	14(2)	24(2)	37(2)	-2(2)	-4(1)	2(2)
C(5A)	18(2)	39(3)	33(2)	-7(2)	-10(1)	11(2)
C(6A)	47(3)	71(4)	32(2)	12(2)	4(2)	13(3)
C(7A)	23(2)	33(2)	29(2)	1(2)	-2(1)	-1(2)
C(8A)	15(2)	18(2)	44(2)	2(2)	-2(2)	-4(2)
C(9A)	19(2)	25(2)	49(2)	5(2)	10(2)	0(2)
C(10A)	36(2)	34(2)	32(2)	1(2)	4(2)	-5(2)
C(11A)	38(3)	41(3)	29(2)	5(2)	-9(2)	-3(2)
C(12A)	23(2)	24(2)	39(2)	-2(2)	-2(2)	-7(2)
N(1B)	26(2)	45(2)	36(2)	-5(2)	0(1)	8(2)
N(2B)	28(2)	74(3)	37(2)	-4(2)	-2(1)	-6(2)
N(3B)	30(2)	50(3)	32(2)	-5(2)	-1(1)	-3(2)
C(4B)	31(2)	29(2)	28(2)	3(2)	-5(2)	-2(2)
C(5B)	35(3)	48(3)	28(2)	8(2)	0(2)	-10(2)
C(6B)	41(3)	84(4)	42(2)	-7(3)	10(2)	-13(3)
C(7B)	18(2)	11(2)	34(2)	3(2)	1(1)	5(2)
C(8B)	30(2)	35(3)	41(2)	0(2)	-3(2)	2(2)
C(9B)	33(3)	48(3)	39(2)	9(2)	4(2)	-2(2)
C(10B)	41(3)	41(3)	28(2)	4(2)	3(2)	15(2)
C(11B)	26(2)	29(2)	39(2)	-3(2)	-6(1)	1(2)
C(12B)	23(2)	33(2)	33(2)	9(2)	4(2)	9(2)

Table S4. Crystallographic data for phtl-ada (**2**) (MFR130)

Table S4a. Atomic coordinates (x 10^4) and equivalent isotropic displacement parameters ($Å^2$ x 10^3) for MFR130. U(eq) is defined as one third of the trace of the orthogonalized U_{ij} tensor.

	x	y	z	U(eq)
N(1)	2998(2)	1551(2)	7750(2)	23(1)
N(2)	4162(2)	1832(2)	7605(2)	30(1)
N(3)	4165(2)	2843(2)	7128(2)	29(1)

C(4)	3004(2)	3213(2)	6967(2)	22(1)
C(5)	2254(2)	2386(2)	7363(2)	22(1)
C(6)	2724(2)	428(2)	8242(2)	22(1)
C(7)	3232(2)	-473(2)	7434(3)	33(1)
C(8)	2961(3)	-1645(2)	7940(3)	35(1)
C(9)	1592(3)	-1788(2)	7981(3)	40(1)
C(10)	1090(2)	-884(2)	8787(3)	33(1)
C(11)	1355(2)	283(2)	8295(3)	30(1)
C(12)	3313(2)	318(2)	9489(2)	30(1)
C(13)	3040(2)	-852(2)	9986(3)	34(1)
C(14)	3552(2)	-1747(2)	9184(3)	37(1)
C(15)	1673(2)	-1002(3)	10035(3)	38(1)
C(16)	2707(2)	4315(2)	6441(2)	24(1)
C(17)	1528(2)	4714(2)	6377(3)	36(1)
C(18)	1259(3)	5758(3)	5888(3)	41(1)
C(19)	2158(3)	6414(2)	5453(3)	39(1)
C(20)	3329(3)	6024(2)	5493(3)	39(1)
C(21)	3603(2)	4982(2)	5984(3)	33(1)

Table S4b. Selected bond lengths [Å] and angles [deg] for MFR130.

N(1)-C(5)	1.346(3)
N(1)-N(2)	1.347(3)
N(1)-C(6)	1.481(3)
N(2)-N(3)	1.315(3)
N(3)-C(4)	1.359(3)
C(4)-C(5)	1.374(3)
C(4)-C(16)	1.466(4)
C(6)-C(12)	1.523(4)
C(6)-C(11)	1.526(3)
C(6)-C(7)	1.529(4)
C(7)-C(8)	1.538(4)
C(8)-C(14)	1.520(4)
C(8)-C(9)	1.526(4)
C(9)-C(10)	1.527(4)
C(10)-C(15)	1.523(4)
C(10)-C(11)	1.526(4)
C(12)-C(13)	1.533(4)
C(13)-C(14)	1.522(4)
C(13)-C(15)	1.525(4)
C(16)-C(17)	1.384(4)
C(16)-C(21)	1.387(4)
C(17)-C(18)	1.382(4)
C(18)-C(19)	1.371(4)
C(19)-C(20)	1.373(4)
C(20)-C(21)	1.383(4)
C(5)-N(1)-N(2)	110.5(2)
C(5)-N(1)-C(6)	130.6(2)
N(2)-N(1)-C(6)	118.87(19)
N(3)-N(2)-N(1)	107.28(19)
N(2)-N(3)-C(4)	109.2(2)
N(3)-C(4)-C(5)	107.8(2)
N(3)-C(4)-C(16)	122.2(2)
C(5)-C(4)-C(16)	130.0(2)
N(1)-C(5)-C(4)	105.2(2)
N(1)-C(6)-C(12)	109.6(2)
N(1)-C(6)-C(11)	109.7(2)
C(12)-C(6)-C(11)	109.4(2)
N(1)-C(6)-C(7)	108.6(2)
C(12)-C(6)-C(7)	109.7(2)
C(11)-C(6)-C(7)	109.8(2)
C(6)-C(7)-C(8)	109.1(2)

C(14)-C(8)-C(9)	110.1(2)
C(14)-C(8)-C(7)	109.4(2)
C(9)-C(8)-C(7)	108.9(2)
C(8)-C(9)-C(10)	109.2(2)
C(15)-C(10)-C(11)	109.8(2)
C(15)-C(10)-C(9)	109.6(2)
C(11)-C(10)-C(9)	109.9(2)
C(10)-C(11)-C(6)	108.9(2)
C(6)-C(12)-C(13)	109.3(2)
C(14)-C(13)-C(15)	109.7(2)
C(14)-C(13)-C(12)	109.2(2)
C(15)-C(13)-C(12)	109.5(2)
C(8)-C(14)-C(13)	109.7(2)
C(10)-C(15)-C(13)	109.2(2)
C(17)-C(16)-C(21)	118.2(2)
C(17)-C(16)-C(4)	121.1(2)
C(21)-C(16)-C(4)	120.6(2)
C(18)-C(17)-C(16)	120.7(3)
C(19)-C(18)-C(17)	120.4(3)
C(18)-C(19)-C(20)	119.7(3)
C(19)-C(20)-C(21)	120.1(3)
C(20)-C(21)-C(16)	120.9(3)

Table S4c. Hydrogen coordinates (x 10^4) and isotropic displacement parameters ($Å^2$ x 10^3) for MFR130.

	x	y	z	U(eq)
H(5)	1403	2399	7362	26
H(7A)	4109	-373	7389	39
H(7B)	2858	-402	6630	39
H(8)	3291	-2232	7422	42
H(9A)	1404	-2538	8286	48
H(9B)	1214	-1718	7178	48
H(10)	203	-983	8819	40
H(11A)	1027	863	8809	36
H(11B)	968	365	7498	36
H(12A)	2997	901	10006	36
H(12B)	4192	422	9462	36
H(13)	3422	-924	10796	41
H(14A)	4430	-1651	9153	45
H(14B)	3395	-2498	9505	45
H(15A)	1493	-1748	10356	46
H(15B)	1344	-432	10558	46
H(17)	904	4271	6669	43
H(18)	455	6021	5854	49
H(19)	1972	7127	5129	47
H(20)	3946	6467	5186	46
H(21)	4408	4721	6008	40

Table S4d. Anisotropic displacement parameters (Å^2 x 10^3) for MFR130.
The anisotropic displacement factor exponent takes the form: $-2 \pi^2 [h^2 a^{*2} U_{11} + ... + 2 h k a^* b^* U_{12}]$

	U_{11}	U_{22}	U_{33}	U_{23}	U_{13}	$U1_2$

N(1)	14(1)	24(1)	31(1)	0(1)	2(1)	-2(1)
N(2)	19(1)	29(1)	43(2)	3(1)	2(1)	-1(1)
N(3)	23(1)	25(1)	39(2)	2(1)	1(1)	-3(1)
C(4)	21(1)	24(2)	21(2)	-3(1)	1(1)	-1(1)
C(5)	17(1)	25(2)	24(2)	0(1)	-2(1)	4(1)
C(6)	21(1)	20(1)	24(2)	1(1)	2(1)	0(1)
C(7)	32(2)	31(2)	36(2)	-3(1)	10(1)	-2(1)
C(8)	39(2)	22(2)	44(2)	-8(2)	12(1)	1(1)
C(9)	42(2)	29(2)	48(2)	-2(2)	-1(2)	-9(1)
C(10)	21(1)	31(2)	47(2)	9(2)	4(1)	-2(1)
C(11)	19(1)	30(2)	41(2)	5(1)	0(1)	0(1)
C(12)	29(2)	30(2)	30(2)	-1(1)	-4(1)	1(1)
C(13)	38(2)	35(2)	28(2)	7(1)	-5(1)	4(1)
C(14)	30(2)	27(2)	55(2)	8(2)	4(2)	5(1)
C(15)	40(2)	33(2)	43(2)	10(2)	12(2)	4(1)
C(16)	26(1)	24(2)	21(2)	-2(1)	2(1)	-2(1)
C(17)	29(2)	35(2)	44(2)	9(2)	10(1)	1(1)
C(18)	39(2)	41(2)	44(2)	13(2)	11(2)	12(2)
C(19)	58(2)	29(2)	30(2)	6(1)	6(2)	3(2)
C(20)	42(2)	36(2)	38(2)	9(2)	2(2)	-12(2)
C(21)	29(2)	39(2)	32(2)	6(2)	0(1)	-6(1)

Table S5. Crystallographic data for **7a** (MFR99B)

Table S5a. Atomic coordinates (x 10^4) and equivalent isotropic displacement parameters ($Å^2$ x 10^3) for MFR99B. U(eq) is defined as one third of the trace of the orthogonalized U_{ij} tensor.

	x	y	z	U(eq)
C(1)	3470(1)	9737(3)	7312(1)	26(1)
C(2)	2241(1)	9087(3)	7372(1)	29(1)
O(1)	1961(1)	9332(2)	8244(1)	34(1)
C(3)	1405(1)	10402(3)	6750(1)	35(1)
C(4)	1626(1)	10328(3)	5791(1)	32(1)
C(5)	1255(1)	8171(3)	5390(1)	37(1)
C(6)	1484(2)	7873(3)	4450(1)	40(1)
N(1)	682(1)	9304(3)	3880(1)	49(1)
N(2)	687(1)	9003(3)	3090(1)	44(1)
N(3)	615(2)	8835(4)	2353(1)	61(1)
C(7)	2710(2)	8407(3)	4365(1)	39(1)
C(8)	3035(2)	10587(3)	4734(1)	37(1)
C(9)	2868(1)	10918(3)	5702(1)	31(1)
C(10)	3702(1)	9551(3)	6344(1)	26(1)
C(11)	4947(1)	10002(3)	6247(1)	38(1)
C(12)	5812(1)	8850(3)	6910(1)	35(1)
C(13)	5591(1)	9212(3)	7856(1)	28(1)
C(14)	6283(1)	7780(3)	8554(1)	29(1)
C(15)	5502(1)	7546(3)	9276(1)	38(1)
C(16)	4306(1)	8262(3)	8884(1)	36(1)
C(17)	4362(1)	8432(3)	7898(1)	26(1)
C(18)	3047(2)	13299(3)	5899(1)	46(1)
C(19)	5746(2)	11568(3)	8118(1)	38(1)
C(20)	7494(1)	8469(3)	8934(1)	32(1)
C(21)	8043(2)	6846(3)	9614(1)	34(1)
C(22)	8274(2)	8803(4)	8229(1)	48(1)
C(23)	8992(1)	7812(3)	10274(1)	33(1)
C(24)	8511(1)	9344(3)	10878(1)	31(1)
O(2)	8781(1)	11345(2)	10761(1)	39(1)
O(3)	7914(1)	8784(2)	11418(1)	41(1)

Table S5b. Selected bond lengths [Å] and angles [deg] for MFR99B.

C(1)-C(17)	1.531(2)
C(1)-C(2)	1.531(2)
C(1)-C(10)	1.5547(19)
C(2)-O(1)	1.4335(17)
C(2)-C(3)	1.524(2)
C(3)-C(4)	1.533(2)
C(4)-C(5)	1.527(3)
C(4)-C(9)	1.546(2)
C(5)-C(6)	1.517(2)
C(6)-N(1)	1.500(2)
C(6)-C(7)	1.520(3)
N(1)-N(2)	1.230(2)
N(2)-N(3)	1.128(2)
C(7)-C(8)	1.510(3)
C(8)-C(9)	1.542(2)
C(9)-C(18)	1.534(3)
C(9)-C(10)	1.556(2)
C(10)-C(11)	1.534(2)
C(11)-C(12)	1.526(2)
C(12)-C(13)	1.530(2)
C(13)-C(19)	1.537(3)
C(13)-C(14)	1.546(2)
C(13)-C(17)	1.551(2)
C(14)-C(20)	1.540(2)
C(14)-C(15)	1.548(2)
C(15)-C(16)	1.534(2)
C(16)-C(17)	1.5291(19)
C(20)-C(22)	1.534(2)
C(20)-C(21)	1.539(2)
C(21)-C(23)	1.535(2)
C(23)-C(24)	1.503(2)
C(24)-O(3)	1.216(2)
C(24)-O(2)	1.315(2)
C(17)-C(1)-C(2)	114.33(13)
C(17)-C(1)-C(10)	108.87(12)
C(2)-C(1)-C(10)	109.48(12)
O(1)-C(2)-C(3)	108.25(13)
O(1)-C(2)-C(1)	111.97(12)
C(3)-C(2)-C(1)	111.34(14)
C(2)-C(3)-C(4)	113.80(14)
C(5)-C(4)-C(3)	109.67(15)
C(5)-C(4)-C(9)	113.79(14)
C(3)-C(4)-C(9)	111.66(14)
C(6)-C(5)-C(4)	114.43(16)
N(1)-C(6)-C(5)	107.37(15)
N(1)-C(6)-C(7)	110.99(16)
C(5)-C(6)-C(7)	110.78(14)
N(2)-N(1)-C(6)	113.30(16)
N(3)-N(2)-N(1)	174.3(2)
C(8)-C(7)-C(6)	111.47(15)
C(7)-C(8)-C(9)	115.09(15)
C(18)-C(9)-C(8)	106.79(15)
C(18)-C(9)-C(4)	108.92(15)
C(8)-C(9)-C(4)	107.68(13)
C(18)-C(9)-C(10)	110.66(14)
C(8)-C(9)-C(10)	112.44(13)
C(4)-C(9)-C(10)	110.21(13)
C(11)-C(10)-C(1)	112.29(12)
C(11)-C(10)-C(9)	112.11(13)
C(1)-C(10)-C(9)	112.57(13)

C(12)-C(11)-C(10)	114.76(14)
C(11)-C(12)-C(13)	112.08(14)
C(12)-C(13)-C(19)	111.18(15)
C(12)-C(13)-C(14)	115.33(14)
C(19)-C(13)-C(14)	110.14(13)
C(12)-C(13)-C(17)	106.20(12)
C(19)-C(13)-C(17)	111.89(14)
C(14)-C(13)-C(17)	101.67(13)
C(20)-C(14)-C(13)	118.73(14)
C(20)-C(14)-C(15)	112.10(12)
C(13)-C(14)-C(15)	103.74(13)
C(16)-C(15)-C(14)	107.81(13)
C(17)-C(16)-C(15)	104.14(13)
C(16)-C(17)-C(1)	119.91(13)
C(16)-C(17)-C(13)	103.49(12)
C(1)-C(17)-C(13)	112.77(13)
C(22)-C(20)-C(21)	109.16(14)
C(22)-C(20)-C(14)	113.31(13)
C(21)-C(20)-C(14)	111.06(14)
C(23)-C(21)-C(20)	113.08(16)
C(24)-C(23)-C(21)	110.67(13)
O(3)-C(24)-O(2)	123.07(17)
O(3)-C(24)-C(23)	122.99(18)
O(2)-C(24)-C(23)	113.93(15)

Symmetry transformations used to generate equivalent atoms: @not found@

Table S5c. Hydrogen coordinates (x 10^4) and isotropic displacement parameters (Å^2 x 10^3) for MFR99B.

	x	y	z	U(eq)
H(1)	3562	11250	7490	32
H(2)	2147	7569	7203	34
H(1A)	1985	10614	8380	50
H(3A)	1442	11888	6948	42
H(3B)	635	9881	6781	42
H(4)	1135	11422	5467	38
H(5A)	1649	7045	5754	45
H(5B)	439	7998	5407	45
H(6)	1329	6373	4272	47
H(7A)	3210	7334	4678	47
H(7B)	2816	8362	3744	47
H(8A)	2584	11656	4375	45
H(8B)	3835	10844	4678	45
H(10)	3558	8046	6170	32
H(11A)	5076	9591	5654	46
H(11B)	5080	11540	6306	46
H(12A)	6576	9358	6847	42
H(12B)	5782	7319	6783	42
H(14)	6340	6357	8285	34
H(15A)	5488	6060	9470	46
H(15B)	5785	8433	9785	46
H(16A)	3736	7212	9003	44
H(16B)	4118	9642	9126	44
H(17)	4315	6959	7665	31
H(18A)	2488	14119	5520	69
H(18B)	3803	13710	5794	69
H(18C)	2964	13573	6508	69
H(19A)	5232	12437	7721	57
H(19B)	6523	11996	8085	57

H(19C)	5581	11759	8714	57
H(20)	7437	9844	9240	39
H(21A)	7457	6260	9935	41
H(21B)	8357	5666	9304	41
H(22A)	8234	7566	7847	72
H(22B)	9049	8997	8510	72
H(22C)	8032	10058	7884	72
H(23A)	9400	6671	10620	40
H(23B)	9534	8560	9956	40
H(2A)	8530(2)	12160(4)	11085(15)	59

Table S5d. Anisotropic displacement parameters ($Å^2 \times 10^3$) for MFR99B.
The anisotropic displacement factor exponent takes the form: $-2 \pi^2 [h^2 a^{*2} U_{11} + ... + 2 h k a^* b^* U_{12}]$

	U11	U22	U33	U23	U13	U12
C(1)	29(1)	24(1)	25(1)	-1(1)	3(1)	-1(1)
C(2)	28(1)	32(1)	26(1)	-4(1)	5(1)	1(1)
O(1)	34(1)	37(1)	31(1)	-1(1)	11(1)	2(1)
C(3)	29(1)	42(1)	35(1)	-1(1)	4(1)	8(1)
C(4)	31(1)	34(1)	28(1)	1(1)	0(1)	9(1)
C(5)	29(1)	45(1)	36(1)	-4(1)	0(1)	-1(1)
C(6)	40(1)	41(1)	36(1)	-6(1)	-3(1)	4(1)
N(1)	43(1)	64(1)	36(1)	-7(1)	-8(1)	12(1)
N(2)	37(1)	53(1)	40(1)	2(1)	-6(1)	-3(1)
N(3)	64(1)	78(2)	37(1)	6(1)	-4(1)	-3(1)
C(7)	39(1)	52(1)	25(1)	0(1)	3(1)	14(1)
C(8)	33(1)	47(1)	30(1)	12(1)	1(1)	3(1)
C(9)	36(1)	27(1)	29(1)	5(1)	1(1)	2(1)
C(10)	28(1)	27(1)	24(1)	0(1)	4(1)	1(1)
C(11)	30(1)	56(1)	28(1)	8(1)	5(1)	-1(1)
C(12)	27(1)	51(1)	28(1)	2(1)	6(1)	2(1)
C(13)	28(1)	30(1)	25(1)	0(1)	3(1)	1(1)
C(14)	29(1)	28(1)	28(1)	-1(1)	2(1)	0(1)
C(15)	32(1)	54(1)	28(1)	6(1)	2(1)	-2(1)
C(16)	31(1)	53(1)	25(1)	4(1)	4(1)	0(1)
C(17)	29(1)	27(1)	23(1)	-1(1)	5(1)	-1(1)
C(18)	57(1)	29(1)	49(1)	9(1)	-4(1)	2(1)
C(19)	35(1)	31(1)	47(1)	0(1)	-1(1)	-3(1)
C(20)	30(1)	35(1)	32(1)	1(1)	1(1)	1(1)
C(21)	33(1)	36(1)	31(1)	-1(1)	0(1)	4(1)
C(22)	30(1)	69(2)	44(1)	13(1)	1(1)	2(1)
C(23)	26(1)	42(1)	31(1)	-1(1)	2(1)	6(1)
C(24)	24(1)	40(1)	28(1)	2(1)	-1(1)	2(1)
O(2)	44(1)	37(1)	39(1)	-2(1)	17(1)	-3(1)
O(3)	45(1)	41(1)	40(1)	3(1)	18(1)	-1(1)

Table S5e. Hydrogen-bonds for MFR99B [Å and deg.].

D-H...A	d(D-H)	d(H...A)	d(D...A)	<(DHA)
O(1)-H(1A)...O(3)#1	0.83	2.01	2.8414(19)	174.0
O(2)-H(2A)...O(1)#1	0.80(3)	1.85(3)	2.6450(18)	172(3)

Symmetry transformations used to generate equivalent atoms:

#1 -x+1,y+1/2,-z+2

Table S6. Crystallographic dara for **6a** (MFR99)

Table S6a. Atomic coordinates (x 10^4) and equivalent isotropic displacement parameters (Å^2 x 10^3) for MFR99. U(eq) is defined as one third of the trace of the orthogonalized U_{ij} tensor.

	x	y	z	U(eq)
C(1)	6878(2)	99(5)	6396(3)	43(1)
C(2)	6842(2)	-1544(6)	6901(3)	54(1)
N(1)	6151(2)	-1625(5)	7023(3)	62(1)
N(2)	6039(2)	-2837(7)	7415(4)	93(2)
C(3)	7386(2)	-1687(6)	7763(3)	63(1)
C(4)	7452(2)	-98(6)	8362(3)	55(1)
C(5)	6905(2)	-20(7)	8848(3)	65(1)
C(6)	6982(3)	1514(9)	9456(4)	83(2)
O(1)	6450(2)	1510(9)	9888(3)	123(2)
C(7)	6967(3)	3114(8)	8943(3)	73(2)
C(8)	7541(2)	3085(7)	8497(3)	66(1)
C(9)	7523(2)	1558(6)	7867(3)	50(1)
C(10)	6932(2)	1688(6)	7005(3)	43(1)
C(11)	6923(3)	3318(6)	6468(3)	59(1)
C(12)	6368(2)	3437(6)	5610(3)	55(1)
C(13)	6361(2)	1903(6)	5004(3)	47(1)
C(14)	5737(2)	1649(6)	4197(3)	50(1)
C(15)	5731(2)	-307(7)	4038(3)	61(1)
C(16)	6151(2)	-1148(7)	4898(3)	63(1)
C(17)	6306(2)	311(5)	5556(3)	45(1)
C(18)	8195(2)	1541(8)	7633(3)	74(2)
C(19)	6997(2)	1878(8)	4670(3)	72(2)
C(20)	5672(2)	2707(7)	3352(3)	60(1)
C(21)	5063(2)	2170(10)	2609(3)	82(2)
C(22)	5650(3)	4643(8)	3539(4)	77(2)
C(23)	5065(3)	2977(12)	1701(4)	107(3)
C(24)	5585(3)	2162(14)	1330(3)	99(3)
O(2)	5763(2)	3217(8)	808(4)	121(2)
O(3)	5798(4)	756(10)	1349(6)	194(4)

Table S6b. Selected bond lengths [Å] and angles [deg] for MFR99.

C(1)-C(2)	1.519(6)
C(1)-C(17)	1.523(6)
C(1)-C(10)	1.550(5)
C(2)-N(1)	1.500(5)
C(2)-C(3)	1.509(6)
N(1)-N(2)	1.184(6)
N(2)-N(3)	1.175(8)
C(3)-C(4)	1.539(7)
C(4)-C(5)	1.524(6)
C(4)-C(9)	1.536(6)
C(5)-C(6)	1.510(8)
C(6)-O(1)	1.440(6)
C(6)-C(7)	1.481(8)
C(7)-C(8)	1.532(7)

C(8)-C(9)	1.539(7)
C(9)-C(18)	1.535(6)
C(9)-C(10)	1.562(5)
C(10)-C(11)	1.522(6)
C(11)-C(12)	1.516(7)
C(12)-C(13)	1.525(6)
C(13)-C(17)	1.535(6)
C(13)-C(19)	1.544(5)
C(13)-C(14)	1.558(6)
C(14)-C(20)	1.528(6)
C(14)-C(15)	1.552(7)
C(15)-C(16)	1.536(7)
C(16)-C(17)	1.510(6)
C(20)-C(21)	1.527(6)
C(20)-C(22)	1.548(8)
C(21)-C(23)	1.546(8)
C(23)-C(24)	1.495(9)
C(24)-O(3)	1.184(10)
C(24)-O(2)	1.280(8)

C(2)-C(1)-C(17)	113.6(3)
C(2)-C(1)-C(10)	111.8(3)
C(17)-C(1)-C(10)	110.4(3)
N(1)-C(2)-C(3)	113.7(4)
N(1)-C(2)-C(1)	106.4(3)
C(3)-C(2)-C(1)	113.1(4)
N(2)-N(1)-C(2)	115.2(4)
N(3)-N(2)-N(1)	170.4(7)
C(2)-C(3)-C(4)	113.7(4)
C(5)-C(4)-C(9)	113.4(4)
C(5)-C(4)-C(3)	112.0(4)
C(9)-C(4)-C(3)	112.7(4)
C(6)-C(5)-C(4)	111.9(4)
O(1)-C(6)-C(7)	110.1(5)
O(1)-C(6)-C(5)	108.8(5)
C(7)-C(6)-C(5)	110.7(4)
C(6)-C(7)-C(8)	108.9(4)
C(7)-C(8)-C(9)	114.1(4)
C(18)-C(9)-C(4)	109.1(4)
C(18)-C(9)-C(8)	106.7(4)
C(4)-C(9)-C(8)	109.1(4)
C(18)-C(9)-C(10)	110.8(4)
C(4)-C(9)-C(10)	109.0(3)
C(8)-C(9)-C(10)	112.0(4)
C(11)-C(10)-C(1)	110.5(3)
C(11)-C(10)-C(9)	114.6(4)
C(1)-C(10)-C(9)	112.5(3)
C(12)-C(11)-C(10)	115.4(4)
C(11)-C(12)-C(13)	112.0(4)
C(12)-C(13)-C(17)	106.6(3)
C(12)-C(13)-C(19)	110.4(4)
C(17)-C(13)-C(19)	112.1(4)
C(12)-C(13)-C(14)	117.8(4)
C(17)-C(13)-C(14)	100.1(3)
C(19)-C(13)-C(14)	109.4(3)
C(20)-C(14)-C(15)	113.8(4)
C(20)-C(14)-C(13)	119.1(3)
C(15)-C(14)-C(13)	102.9(4)
C(16)-C(15)-C(14)	107.9(4)
C(17)-C(16)-C(15)	103.5(4)
C(16)-C(17)-C(1)	118.3(4)
C(16)-C(17)-C(13)	105.7(3)
C(1)-C(17)-C(13)	114.2(3)
C(21)-C(20)-C(14)	111.5(4)
C(21)-C(20)-C(22)	110.6(5)
C(14)-C(20)-C(22)	111.8(4)

C(20)-C(21)-C(23)	111.9(5)
C(24)-C(23)-C(21)	110.7(6)
O(3)-C(24)-O(2)	116.5(6)
O(3)-C(24)-C(23)	133.8(9)
O(2)-C(24)-C(23)	109.2(8)

Table S6c. Hydrogen coordinates (x 10^4) and isotropic displacement parameters (Å^2 x 10^3) for MFR99.

	x	y	z	U(eq)
H(1)	7295	49	6203	52
H(2)	6893	-2510	6514	65
H(3A)	7812	-1886	7622	76
H(3B)	7295	-2681	8095	76
H(4)	7876	-237	8832	66
H(5A)	6469	30	8408	78
H(5B)	6919	-1061	9202	78
H(6)	7415	1435	9916	99
H(1A)	6258	572	9810	184
H(7A)	7015	4099	9344	87
H(7B)	6540	3214	8488	87
H(8A)	7965	3062	8963	79
H(8B)	7530	4143	8157	79
H(10)	6519	1705	7209	52
H(11A)	6885	4293	6847	70
H(11B)	7351	3418	6321	70
H(12A)	6425	4482	5292	66
H(12B)	5938	3512	5755	66
H(14)	5342	1912	4414	61
H(15A)	5273	-740	3894	73
H(15B)	5920	-568	3539	73
H(16A)	5898	-2045	5102	76
H(16B)	6562	-1642	4807	76
H(17)	5900	476	5764	54
H(18A)	8236	494	7318	110
H(18B)	8556	1605	8177	110
H(18C)	8220	2512	7256	110
H(19A)	7385	1728	5173	108
H(19B)	7035	2949	4373	108
H(19C)	6971	943	4253	108
H(20)	6073	2488	3139	72
H(21A)	4657	2517	2768	99
H(21B)	5058	924	2553	99
H(22A)	5297	4875	3824	115
H(22B)	5564	5267	2981	115
H(22C)	6075	5001	3929	115
H(23A)	4625	2833	1279	128
H(23B)	5155	4203	1780	128
H(2A)	6135	2941	752	181

Table S6d. Anisotropic displacement parameters (Å^2 x 10^3) for MFR99. The anisotropic displacement factor exponent takes the form: $-2 \pi^2 [h^2 a^{*2} U_{11} + ... + 2 h k a^* b^* U_{12}]$

	U_{11}	U_{22}	U_{33}	U_{23}	U_{13}	U_{12}
C(1)	39(2)	36(3)	58(3)	-1(2)	19(2)	2(2)
C(2)	52(3)	32(3)	75(3)	-2(2)	9(2)	3(2)
N(1)	55(2)	42(2)	88(3)	22(2)	20(2)	-9(2)
N(2)	61(3)	63(3)	146(5)	27(3)	13(3)	-12(2)
N(3)	104(4)	113(5)	266(9)	115(6)	39(5)	-19(4)
C(3)	53(3)	40(3)	92(4)	9(3)	13(3)	11(2)
C(4)	43(2)	52(3)	67(3)	2(3)	8(2)	3(2)
C(5)	54(3)	82(4)	55(3)	18(3)	9(2)	6(3)
C(6)	81(4)	108(5)	66(3)	4(4)	31(3)	15(4)
O(1)	96(3)	205(6)	83(3)	0(4)	51(2)	12(3)
C(7)	74(3)	85(4)	56(3)	-20(3)	11(2)	33(3)
C(8)	72(3)	54(3)	60(3)	-8(3)	0(2)	-3(3)
C(9)	36(2)	47(3)	65(3)	-5(3)	10(2)	-9(2)
C(10)	44(2)	38(2)	52(2)	-8(2)	22(2)	0(2)
C(11)	85(3)	35(3)	56(3)	-5(2)	19(3)	-9(2)
C(12)	71(3)	33(2)	65(3)	1(2)	23(2)	-12(2)
C(13)	44(2)	45(3)	61(3)	-7(2)	28(2)	-12(2)
C(14)	36(2)	64(3)	58(3)	-12(3)	23(2)	0(2)
C(15)	52(3)	71(3)	65(3)	-25(3)	26(2)	-6(2)
C(16)	53(3)	55(3)	79(4)	-20(3)	11(2)	0(2)
C(17)	37(2)	36(3)	65(3)	-4(2)	23(2)	-2(2)
C(18)	45(2)	86(4)	92(4)	-8(3)	23(2)	-3(3)
C(19)	47(3)	104(4)	72(3)	-19(3)	27(2)	-22(3)
C(20)	42(2)	92(4)	54(3)	7(3)	26(2)	-6(2)
C(21)	45(3)	135(6)	69(3)	-6(3)	18(2)	-8(3)
C(22)	85(4)	84(4)	64(3)	21(3)	24(3)	-2(3)
C(23)	75(4)	174(8)	76(4)	-1(5)	28(3)	17(4)
C(24)	73(4)	207(9)	32(3)	10(4)	38(3)	0(5)
O(2)	83(3)	125(4)	151(5)	-14(4)	25(3)	11(3)
O(3)	174(6)	115(5)	308(11)	66(6)	87(6)	52(5)

Table S6e. Hydrogen-bonds for MFR99 [Å and deg.].

D-H...A	d(D-H)	d(H...A)	d(D...A)	<(DHA)
O(2)-H(2A)...O(1)#1	0.83	1.99	2.638(7)	134.6

Symmetry transformations used to generate equivalent atoms:

#1 x,y,z-1

Table S7. Crystallographic data for phtl-DC (PYTLUD)

Table S7a. Atomic coordinates (x 10^4) and equivalent isotropic displacement parameters ($Å^2$ x 10^3) for PYTLUD. U(eq) is defined as one third of the trace of the orthogonalized U_{ij} tensor.

	x	y	z	U(eq)
O(1A)	5671(2)	-4523(2)	753(1)	67(1)
O(2A)	3956(2)	538(2)	1876(1)	53(1)
O(3A)	3051(2)	-804(2)	2060(1)	55(1)

N(1A)	3136(2)	-2056(2)	-99(1)	47(1)
N(2A)	2558(2)	-1289(2)	-218(1)	60(1)
N(3A)	2555(2)	-1701(3)	-408(1)	58(1)
N(4A)	4151(2)	-4254(2)	-588(1)	49(1)
C(1A)	3323(3)	-1768(3)	120(1)	52(1)
C(2A)	4206(3)	-2579(3)	226(1)	47(1)
C(3A)	3710(2)	-3748(3)	296(1)	44(1)
C(4A)	4645(3)	-4375(3)	428(1)	47(1)
C(5A)	4818(2)	-3847(3)	639(1)	45(1)
C(6A)	3650(2)	-3774(3)	761(1)	40(1)
C(7A)	3748(2)	-3174(3)	970(1)	36(1)
C(8A)	4652(2)	-3602(3)	1130(1)	47(1)
C(9A)	4195(3)	-3096(3)	1336(1)	52(1)
C(10A)	2965(2)	-2528(3)	1294(1)	40(1)
C(11A)	2552(2)	-3112(3)	1090(1)	37(1)
C(12A)	1675(3)	-2422(3)	959(1)	48(1)
C(13A)	1515(2)	-2950(3)	745(1)	55(1)
C(14A)	2702(2)	-3134(3)	629(1)	43(1)
C(15A)	2506(3)	-3662(3)	411(1)	47(1)
C(16A)	1658(3)	-2923(4)	277(1)	68(1)
C(17A)	2128(3)	-1727(3)	232(1)	67(1)
C(18A)	2057(3)	-4317(3)	1138(1)	55(1)
C(19A)	1946(3)	-4874(3)	428(1)	77(1)
C(20A)	3511(3)	-2949(3)	-213(1)	48(1)
C(21A)	3147(3)	-2731(3)	-409(1)	42(1)
C(22A)	3381(3)	-3360(3)	-600(1)	40(1)
C(23A)	2868(3)	-3039(3)	-786(1)	48(1)
C(24A)	3185(3)	-3619(3)	-964(1)	51(1)
C(25A)	3988(3)	-4516(3)	-951(1)	56(1)
C(26A)	4435(3)	-4808(3)	-762(1)	58(1)
C(27A)	2111(3)	-2569(3)	1481(1)	51(1)
C(28A)	906(3)	-1971(3)	1440(1)	66(1)
C(29A)	2649(3)	-2130(3)	1683(1)	56(1)
C(30A)	2963(3)	-892(3)	1682(1)	55(1)
C(31A)	3312(3)	-405(3)	1895(1)	46(1)
O(1B)	2046(2)	1137(2)	970(1)	51(1)
O(2B)	4950(2)	4818(2)	-237(1)	59(1)
O(3B)	4971(2)	3152(2)	-402(1)	74(1)
N(1B)	5466(2)	3710(2)	1712(1)	39(1)
N(2B)	5555(2)	4663(2)	1827(1)	50(1)
N(3B)	5551(2)	4349(2)	2024(1)	50(1)
N(4B)	4741(2)	1555(2)	2222(1)	37(1)
C(1B)	5408(3)	3833(3)	1486(1)	43(1)
C(2B)	4279(2)	3272(2)	1399(1)	42(1)
C(3B)	4325(2)	1955(2)	1377(1)	36(1)
C(4B)	3182(2)	1503(3)	1278(1)	42(1)
C(5B)	3067(2)	1749(3)	1049(1)	38(1)
C(6B)	4179(2)	1373(3)	930(1)	36(1)
C(7B)	4167(2)	1751(2)	706(1)	35(1)
C(8B)	3084(2)	1483(3)	569(1)	44(1)
C(9B)	3557(2)	1667(3)	348(1)	47(1)
C(10B)	4943(2)	1809(3)	362(1)	38(1)
C(11B)	5246(2)	1320(2)	578(1)	38(1)
C(12B)	6375(2)	1812(3)	678(1)	47(1)
C(13B)	6456(2)	1506(3)	906(1)	45(1)
C(14B)	5342(2)	1839(3)	1031(1)	36(1)
C(15B)	5457(2)	1528(2)	1263(1)	35(1)
C(16B)	6556(2)	2077(3)	1366(1)	46(1)
C(17B)	6524(3)	3382(3)	1381(1)	50(1)
C(18B)	5290(3)	-6(2)	572(1)	51(1)
C(19B)	5578(3)	209(2)	1292(1)	51(1)
C(20B)	5395(3)	2780(3)	1835(1)	41(1)
C(21B)	5446(2)	3177(3)	2031(1)	34(1)
C(22B)	5384(2)	2547(3)	2227(1)	32(1)
C(23B)	5929(2)	2945(3)	2404(1)	40(1)
C(24B)	5820(3)	2317(3)	2582(1)	47(1)

C(25B)	5150(3)	1317(3)	2583(1)	48(1)
C(26B)	4638(3)	968(3)	2400(1)	43(1)
C(27B)	5595(3)	1332(3)	172(1)	44(1)
C(28B)	6971(3)	1421(3)	188(1)	61(1)
C(29B)	5148(3)	1900(2)	-27(1)	42(1)
C(30B)	5365(3)	3188(3)	-40(1)	48(1)
C(31B)	5075(3)	3683(3)	-245(1)	45(1)
O(1C)	3175(2)	56(2)	7524(1)	62(1)
O(2C)	6317(2)	4441(2)	6490(1)	59(1)
O(3C)	7444(2)	3159(2)	6331(1)	68(1)
N(1C)	5149(2)	2696(2)	8405(1)	43(1)
N(2C)	5239(2)	3628(2)	8525(1)	45(1)
N(3C)	5052(2)	3290(2)	8716(1)	45(1)
N(4C)	4022(2)	453(2)	8874(1)	41(1)
C(1C)	5346(3)	2830(3)	8181(1)	50(1)
C(2C)	4444(3)	2151(3)	8053(1)	48(1)
C(3C)	4704(2)	875(2)	8018(1)	40(1)
C(4C)	3806(3)	362(3)	7863(1)	50(1)
C(5C)	4016(2)	730(3)	7641(1)	44(1)
C(6C)	5315(2)	547(2)	7572(1)	36(1)
C(7C)	5575(2)	1075(2)	7361(1)	33(1)
C(8C)	4813(3)	750(3)	7175(1)	49(1)
C(9C)	5557(2)	1116(3)	6988(1)	49(1)
C(10C)	6802(2)	1481(2)	7069(1)	35(1)
C(11C)	6870(2)	883(2)	7282(1)	33(1)
C(12C)	7713(2)	1431(3)	7439(1)	43(1)
C(13C)	7498(2)	957(3)	7656(1)	48(1)
C(14C)	6194(2)	1082(3)	7728(1)	34(1)
C(15C)	6009(2)	637(2)	7952(1)	37(1)
C(16C)	6833(3)	1240(3)	8108(1)	47(1)
C(17C)	6643(3)	2531(3)	8123(1)	58(1)
C(18C)	7166(3)	-400(3)	7254(1)	52(1)
C(19C)	6273(3)	-660(3)	7970(1)	59(1)
C(20C)	4896(3)	1765(3)	8518(1)	49(1)
C(21C)	4834(2)	2136(3)	8717(1)	38(1)
C(22C)	4517(2)	1494(3)	8903(1)	37(1)
C(23C)	4690(3)	1918(3)	9100(1)	50(1)
C(24C)	4335(3)	1275(3)	9268(1)	55(1)
C(25C)	3825(3)	227(3)	9237(1)	52(1)
C(26C)	3691(3)	-151(3)	9038(1)	49(1)
C(27C)	7810(3)	1288(3)	6911(1)	42(1)
C(28C)	9016(2)	1764(3)	6985(1)	62(1)
C(29C)	7500(3)	1778(3)	6698(1)	43(1)
C(30C)	7254(3)	3070(3)	6699(1)	46(1)
C(31C)	7030(3)	3537(3)	6488(1)	41(1)

Table S7b. Selected bond lengths [Å] and angles [deg] for PYTLUD.

O(1A)-C(5A)	1.432(3)
O(2A)-C(31A)	1.310(4)
O(3A)-C(31A)	1.201(4)
N(1A)-C(20A)	1.336(4)
N(1A)-N(2A)	1.340(3)
N(1A)-C(1A)	1.477(4)
N(2A)-N(3A)	1.316(3)
N(3A)-C(21A)	1.362(4)
N(4A)-C(26A)	1.334(4)
N(4A)-C(22A)	1.347(4)
C(1A)-C(17A)	1.514(4)
C(1A)-C(2A)	1.521(4)
C(2A)-C(3A)	1.531(4)
C(3A)-C(4A)	1.531(4)

C(3A)-C(15A)	1.536(4)
C(4A)-C(5A)	1.510(4)
C(5A)-C(6A)	1.521(4)
C(6A)-C(7A)	1.527(4)
C(6A)-C(14A)	1.547(4)
C(7A)-C(8A)	1.526(4)
C(7A)-C(11A)	1.545(4)
C(8A)-C(9A)	1.543(4)
C(9A)-C(10A)	1.542(4)
C(10A)-C(27A)	1.542(4)
C(10A)-C(11A)	1.551(4)
C(11A)-C(12A)	1.522(4)
C(11A)-C(18A)	1.531(4)
C(12A)-C(13A)	1.523(4)
C(13A)-C(14A)	1.535(4)
C(14A)-C(15A)	1.552(4)
C(15A)-C(19A)	1.539(4)
C(15A)-C(16A)	1.542(4)
C(16A)-C(17A)	1.508(5)
C(20A)-C(21A)	1.355(4)
C(21A)-C(22A)	1.460(4)
C(22A)-C(23A)	1.385(4)
C(23A)-C(24A)	1.381(4)
C(24A)-C(25A)	1.372(4)
C(25A)-C(26A)	1.364(4)
C(27A)-C(29A)	1.527(4)
C(27A)-C(28A)	1.533(4)
C(29A)-C(30A)	1.475(4)
C(30A)-C(31A)	1.537(4)
O(1B)-C(5B)	1.433(3)
O(2B)-C(31B)	1.323(4)
O(3B)-C(31B)	1.193(3)
N(1B)-N(2B)	1.333(3)
N(1B)-C(20B)	1.341(3)
N(1B)-C(1B)	1.470(3)
N(2B)-N(3B)	1.326(3)
N(3B)-C(21B)	1.363(4)
N(4B)-C(26B)	1.339(3)
N(4B)-C(22B)	1.354(3)
C(1B)-C(17B)	1.510(4)
C(1B)-C(2B)	1.523(4)
C(2B)-C(3B)	1.532(4)
C(3B)-C(4B)	1.517(4)
C(3B)-C(15B)	1.544(4)
C(4B)-C(5B)	1.517(4)
C(5B)-C(6B)	1.520(4)
C(6B)-C(7B)	1.520(4)
C(6B)-C(14B)	1.547(4)
C(7B)-C(8B)	1.526(4)
C(7B)-C(11B)	1.541(4)
C(8B)-C(9B)	1.545(4)
C(9B)-C(10B)	1.553(4)
C(10B)-C(27B)	1.534(4)
C(10B)-C(11B)	1.547(4)
C(11B)-C(12B)	1.524(4)
C(11B)-C(18B)	1.536(4)
C(12B)-C(13B)	1.523(4)
C(13B)-C(14B)	1.529(4)
C(14B)-C(15B)	1.547(4)
C(15B)-C(16B)	1.532(4)
C(15B)-C(19B)	1.545(4)
C(16B)-C(17B)	1.514(4)
C(20B)-C(21B)	1.356(4)
C(21B)-C(22B)	1.462(4)
C(22B)-C(23B)	1.378(4)
C(23B)-C(24B)	1.371(4)

C(24B)-C(25B)	1.377(4)
C(25B)-C(26B)	1.376(4)
C(27B)-C(29B)	1.530(4)
C(27B)-C(28B)	1.538(4)
C(29B)-C(30B)	1.512(4)
C(30B)-C(31B)	1.487(4)
O(1C)-C(5C)	1.435(3)
O(2C)-C(31C)	1.313(3)
O(3C)-C(31C)	1.195(3)
N(1C)-C(20C)	1.333(4)
N(1C)-N(2C)	1.335(3)
N(1C)-C(1C)	1.472(4)
N(2C)-N(3C)	1.315(3)
N(3C)-C(21C)	1.357(4)
N(4C)-C(26C)	1.325(4)
N(4C)-C(22C)	1.338(4)
C(1C)-C(2C)	1.524(4)
C(1C)-C(17C)	1.531(4)
C(2C)-C(3C)	1.522(4)
C(3C)-C(4C)	1.538(4)
C(3C)-C(15C)	1.539(4)
C(4C)-C(5C)	1.514(4)
C(5C)-C(6C)	1.528(4)
C(6C)-C(7C)	1.526(4)
C(6C)-C(14C)	1.538(4)
C(7C)-C(8C)	1.523(4)
C(7C)-C(11C)	1.546(4)
C(8C)-C(9C)	1.527(4)
C(9C)-C(10C)	1.542(4)
C(10C)-C(27C)	1.533(4)
C(10C)-C(11C)	1.543(4)
C(11C)-C(12C)	1.526(4)
C(11C)-C(18C)	1.532(4)
C(12C)-C(13C)	1.528(4)
C(13C)-C(14C)	1.531(4)
C(14C)-C(15C)	1.550(4)
C(15C)-C(16C)	1.532(4)
C(15C)-C(19C)	1.535(4)
C(16C)-C(17C)	1.513(4)
C(20C)-C(21C)	1.360(4)
C(21C)-C(22C)	1.463(4)
C(22C)-C(23C)	1.379(4)
C(23C)-C(24C)	1.380(4)
C(24C)-C(25C)	1.355(4)
C(25C)-C(26C)	1.367(4)
C(27C)-C(28C)	1.525(4)
C(27C)-C(29C)	1.534(4)
C(29C)-C(30C)	1.520(4)
C(30C)-C(31C)	1.493(4)

C(20A)-N(1A)-N(2A)	110.2(3)
C(20A)-N(1A)-C(1A)	131.5(3)
N(2A)-N(1A)-C(1A)	118.1(3)
N(3A)-N(2A)-N(1A)	107.3(3)
N(2A)-N(3A)-C(21A)	108.8(3)
C(26A)-N(4A)-C(22A)	118.1(3)
N(1A)-C(1A)-C(17A)	110.0(3)
N(1A)-C(1A)-C(2A)	112.6(3)
C(17A)-C(1A)-C(2A)	111.8(3)
C(1A)-C(2A)-C(3A)	116.5(3)
C(2A)-C(3A)-C(4A)	109.9(2)
C(2A)-C(3A)-C(15A)	113.6(2)
C(4A)-C(3A)-C(15A)	110.6(3)
C(5A)-C(4A)-C(3A)	113.7(2)
O(1A)-C(5A)-C(4A)	109.1(2)
O(1A)-C(5A)-C(6A)	109.4(2)

C(4A)-C(5A)-C(6A)	112.4(2)
C(5A)-C(6A)-C(7A)	115.1(2)
C(5A)-C(6A)-C(14A)	108.9(2)
C(7A)-C(6A)-C(14A)	108.7(2)
C(8A)-C(7A)-C(6A)	120.1(2)
C(8A)-C(7A)-C(11A)	103.9(2)
C(6A)-C(7A)-C(11A)	114.1(2)
C(7A)-C(8A)-C(9A)	104.2(2)
C(10A)-C(9A)-C(8A)	107.6(2)
C(27A)-C(10A)-C(9A)	113.3(3)
C(27A)-C(10A)-C(11A)	118.2(2)
C(9A)-C(10A)-C(11A)	103.1(2)
C(12A)-C(11A)-C(18A)	111.2(2)
C(12A)-C(11A)-C(7A)	107.1(2)
C(18A)-C(11A)-C(7A)	111.7(2)
C(12A)-C(11A)-C(10A)	115.9(2)
C(18A)-C(11A)-C(10A)	109.4(3)
C(7A)-C(11A)-C(10A)	101.2(2)
C(11A)-C(12A)-C(13A)	111.9(2)
C(12A)-C(13A)-C(14A)	113.7(2)
C(13A)-C(14A)-C(6A)	112.4(2)
C(13A)-C(14A)-C(15A)	112.4(2)
C(6A)-C(14A)-C(15A)	114.1(2)
C(3A)-C(15A)-C(19A)	109.1(3)
C(3A)-C(15A)-C(16A)	107.2(3)
C(19A)-C(15A)-C(16A)	107.3(3)
C(3A)-C(15A)-C(14A)	110.1(2)
C(19A)-C(15A)-C(14A)	110.7(3)
C(16A)-C(15A)-C(14A)	112.3(3)
C(17A)-C(16A)-C(15A)	114.0(3)
C(16A)-C(17A)-C(1A)	111.6(3)
N(1A)-C(20A)-C(21A)	106.2(3)
C(20A)-C(21A)-N(3A)	107.6(3)
C(20A)-C(21A)-C(22A)	130.3(3)
N(3A)-C(21A)-C(22A)	121.9(3)
N(4A)-C(22A)-C(23A)	121.2(3)
N(4A)-C(22A)-C(21A)	116.6(3)
C(23A)-C(22A)-C(21A)	122.1(3)
C(24A)-C(23A)-C(22A)	119.5(3)
C(25A)-C(24A)-C(23A)	118.7(3)
C(26A)-C(25A)-C(24A)	118.9(3)
N(4A)-C(26A)-C(25A)	123.5(3)
C(29A)-C(27A)-C(28A)	110.1(3)
C(29A)-C(27A)-C(10A)	115.1(2)
C(28A)-C(27A)-C(10A)	112.8(3)
C(30A)-C(29A)-C(27A)	114.3(3)
C(29A)-C(30A)-C(31A)	114.3(3)
O(3A)-C(31A)-O(2A)	122.3(3)
O(3A)-C(31A)-C(30A)	126.5(3)
O(2A)-C(31A)-C(30A)	111.2(3)
N(2B)-N(1B)-C(20B)	109.8(2)
N(2B)-N(1B)-C(1B)	118.5(2)
C(20B)-N(1B)-C(1B)	131.6(3)
N(3B)-N(2B)-N(1B)	108.0(2)
N(2B)-N(3B)-C(21B)	108.0(3)
C(26B)-N(4B)-C(22B)	117.3(3)
N(1B)-C(1B)-C(17B)	112.4(2)
N(1B)-C(1B)-C(2B)	111.3(2)
C(17B)-C(1B)-C(2B)	111.3(2)
C(1B)-C(2B)-C(3B)	115.6(2)
C(4B)-C(3B)-C(2B)	110.8(2)
C(4B)-C(3B)-C(15B)	111.7(2)
C(2B)-C(3B)-C(15B)	113.0(2)
C(5B)-C(4B)-C(3B)	114.8(2)
O(1B)-C(5B)-C(4B)	108.8(2)
O(1B)-C(5B)-C(6B)	108.9(2)

C(4B)-C(5B)-C(6B)	111.9(2)
C(5B)-C(6B)-C(7B)	113.3(2)
C(5B)-C(6B)-C(14B)	111.5(2)
C(7B)-C(6B)-C(14B)	108.2(2)
C(6B)-C(7B)-C(8B)	120.2(2)
C(6B)-C(7B)-C(11B)	114.4(2)
C(8B)-C(7B)-C(11B)	103.8(2)
C(7B)-C(8B)-C(9B)	104.0(2)
C(8B)-C(9B)-C(10B)	107.2(2)
C(27B)-C(10B)-C(11B)	119.5(2)
C(27B)-C(10B)-C(9B)	112.5(2)
C(11B)-C(10B)-C(9B)	103.4(2)
C(12B)-C(11B)-C(18B)	111.0(2)
C(12B)-C(11B)-C(7B)	107.0(2)
C(18B)-C(11B)-C(7B)	111.3(2)
C(12B)-C(11B)-C(10B)	115.4(2)
C(18B)-C(11B)-C(10B)	110.4(2)
C(7B)-C(11B)-C(10B)	101.4(2)
C(13B)-C(12B)-C(11B)	112.1(2)
C(12B)-C(13B)-C(14B)	114.0(2)
C(13B)-C(14B)-C(6B)	111.5(2)
C(13B)-C(14B)-C(15B)	112.7(2)
C(6B)-C(14B)-C(15B)	113.4(2)
C(16B)-C(15B)-C(3B)	107.9(2)
C(16B)-C(15B)-C(19B)	106.6(2)
C(3B)-C(15B)-C(19B)	109.2(2)
C(16B)-C(15B)-C(14B)	113.1(2)
C(3B)-C(15B)-C(14B)	108.9(2)
C(19B)-C(15B)-C(14B)	111.0(2)
C(17B)-C(16B)-C(15B)	115.0(3)
C(1B)-C(17B)-C(16B)	113.2(3)
N(1B)-C(20B)-C(21B)	106.4(3)
C(20B)-C(21B)-N(3B)	107.8(3)
C(20B)-C(21B)-C(22B)	130.0(3)
N(3B)-C(21B)-C(22B)	122.2(3)
N(4B)-C(22B)-C(23B)	122.2(3)
N(4B)-C(22B)-C(21B)	115.5(3)
C(23B)-C(22B)-C(21B)	122.3(3)
C(24B)-C(23B)-C(22B)	119.1(3)
C(23B)-C(24B)-C(25B)	119.6(3)
C(26B)-C(25B)-C(24B)	118.1(3)
N(4B)-C(26B)-C(25B)	123.7(3)
C(29B)-C(27B)-C(10B)	111.6(2)
C(29B)-C(27B)-C(28B)	110.7(2)
C(10B)-C(27B)-C(28B)	113.1(2)
C(30B)-C(29B)-C(27B)	114.6(2)
C(31B)-C(30B)-C(29B)	113.1(3)
O(3B)-C(31B)-O(2B)	122.5(3)
O(3B)-C(31B)-C(30B)	125.9(3)
O(2B)-C(31B)-C(30B)	111.6(3)
C(20C)-N(1C)-N(2C)	110.4(2)
C(20C)-N(1C)-C(1C)	131.0(3)
N(2C)-N(1C)-C(1C)	118.6(3)
N(3C)-N(2C)-N(1C)	107.3(2)
N(2C)-N(3C)-C(21C)	108.8(3)
C(26C)-N(4C)-C(22C)	118.4(3)
N(1C)-C(1C)-C(2C)	112.7(3)
N(1C)-C(1C)-C(17C)	111.0(3)
C(2C)-C(1C)-C(17C)	111.7(3)
C(3C)-C(2C)-C(1C)	117.1(2)
C(2C)-C(3C)-C(4C)	110.4(2)
C(2C)-C(3C)-C(15C)	113.2(2)
C(4C)-C(3C)-C(15C)	111.2(2)
C(5C)-C(4C)-C(3C)	114.2(2)
O(1C)-C(5C)-C(4C)	104.3(2)
O(1C)-C(5C)-C(6C)	112.7(2)

C(4C)-C(5C)-C(6C)	112.7(2)
C(7C)-C(6C)-C(5C)	112.8(2)
C(7C)-C(6C)-C(14C)	107.9(2)
C(5C)-C(6C)-C(14C)	110.6(2)
C(8C)-C(7C)-C(6C)	120.4(2)
C(8C)-C(7C)-C(11C)	102.7(2)
C(6C)-C(7C)-C(11C)	114.7(2)
C(7C)-C(8C)-C(9C)	105.0(2)
C(8C)-C(9C)-C(10C)	107.0(2)
C(27C)-C(10C)-C(9C)	112.9(2)
C(27C)-C(10C)-C(11C)	119.5(2)
C(9C)-C(10C)-C(11C)	103.1(2)
C(12C)-C(11C)-C(18C)	110.3(2)
C(12C)-C(11C)-C(10C)	116.2(2)
C(18C)-C(11C)-C(10C)	110.1(2)
C(12C)-C(11C)-C(7C)	106.9(2)
C(18C)-C(11C)-C(7C)	112.2(2)
C(10C)-C(11C)-C(7C)	100.8(2)
C(11C)-C(12C)-C(13C)	111.7(2)
C(12C)-C(13C)-C(14C)	113.3(2)
C(13C)-C(14C)-C(6C)	111.3(2)
C(13C)-C(14C)-C(15C)	112.3(2)
C(6C)-C(14C)-C(15C)	113.4(2)
C(16C)-C(15C)-C(19C)	106.3(2)
C(16C)-C(15C)-C(3C)	107.4(2)
C(19C)-C(15C)-C(3C)	109.5(2)
C(16C)-C(15C)-C(14C)	112.6(2)
C(19C)-C(15C)-C(14C)	111.8(3)
C(3C)-C(15C)-C(14C)	109.1(2)
C(17C)-C(16C)-C(15C)	114.3(3)
C(16C)-C(17C)-C(1C)	111.8(3)
N(1C)-C(20C)-C(21C)	106.0(3)
N(3C)-C(21C)-C(20C)	107.5(3)
N(3C)-C(21C)-C(22C)	122.9(3)
C(20C)-C(21C)-C(22C)	129.4(3)
N(4C)-C(22C)-C(23C)	120.6(3)
N(4C)-C(22C)-C(21C)	116.1(3)
C(23C)-C(22C)-C(21C)	123.3(3)
C(22C)-C(23C)-C(24C)	119.9(3)
C(25C)-C(24C)-C(23C)	118.9(3)
C(24C)-C(25C)-C(26C)	118.4(3)
N(4C)-C(26C)-C(25C)	123.8(3)
C(28C)-C(27C)-C(10C)	112.6(2)
C(28C)-C(27C)-C(29C)	110.2(2)
C(10C)-C(27C)-C(29C)	112.5(2)
C(30C)-C(29C)-C(27C)	113.6(3)
C(31C)-C(30C)-C(29C)	112.5(3)
O(3C)-C(31C)-O(2C)	122.4(3)
O(3C)-C(31C)-C(30C)	125.4(3)
O(2C)-C(31C)-C(30C)	112.2(3)

Table S7c. Hydrogen coordinates (x 10^4) and isotropic displacement parameters ($Å^2$ x 10^3) for PYTLUD.

	x	y	z	U(eq)
H(1A)	6328	-4513	692	101
H(2A)	4092	811	1992	80
H(1A1)	3671	-981	126	63
H(2A1)	4534	-2182	347	56

H(2A2)	4875	-2721	131	56
H(3A)	3574	-4220	171	52
H(4A1)	4398	-5182	445	57
H(4A2)	5417	-4370	355	57
H(5A)	5144	-3057	622	54
H(6A)	3358	-4571	785	48
H(7A)	3973	-2364	940	44
H(8A1)	5462	-3319	1098	56
H(8A2)	4667	-4448	1135	56
H(9A1)	4111	-3711	1439	62
H(9A2)	4764	-2522	1388	62
H(10A)	3116	-1704	1263	47
H(12A)	1970	-1629	945	58
H(12B)	893	-2392	1029	58
H(13A)	996	-2444	663	65
H(13B)	1107	-3696	760	65
H(14A)	3033	-2352	605	51
H(16A)	1521	-3325	146	81
H(16B)	882	-2855	347	81
H(17A)	2222	-1304	362	81
H(17B)	1542	-1311	147	81
H(18A)	1288	-4246	1208	82
H(18B)	2617	-4726	1227	82
H(18C)	1952	-4743	1011	82
H(19A)	1976	-5250	294	115
H(19B)	1117	-4808	472	115
H(19C)	2393	-5329	527	115
H(20A)	3942	-3598	-167	57
H(23A)	2309	-2431	-791	58
H(24A)	2857	-3404	-1092	61
H(25A)	4225	-4921	-1070	67
H(26A)	4972	-5434	-754	70
H(27A)	1923	-3395	1504	61
H(28A)	468	-1888	1568	99
H(28B)	438	-2433	1344	99
H(28C)	1049	-1215	1380	99
H(29A)	2072	-2270	1795	67
H(29B)	3374	-2578	1714	67
H(30A)	2275	-453	1629	66
H(30B)	3635	-773	1587	66
H(1B)	1506	1130	1059	77
H(2B)	4802	5071	-354	88
H(1B1)	5357	4670	1456	52
H(2B1)	4119	3606	1263	51
H(2B2)	3601	3473	1489	51
H(3B)	4359	1632	1519	43
H(4B1)	3145	665	1299	50
H(4B2)	2491	1845	1349	50
H(5B)	2943	2588	1029	46
H(6B)	4214	519	933	44
H(7B)	4234	2604	708	42
H(8B1)	2416	2009	599	53
H(8B2)	2813	686	589	53
H(9B1)	3195	2360	287	56
H(9B2)	3354	1002	261	56
H(10B)	5109	2649	367	46
H(12C)	7084	1510	607	57
H(12D)	6375	2654	663	57
H(13C)	7157	1895	966	54
H(13D)	6585	671	920	54
H(14B)	5288	2692	1025	43
H(16C)	6633	1759	1505	55
H(16D)	7275	1851	1288	55
H(17C)	7232	3646	1457	60
H(17D)	6567	3708	1241	60
H(18D)	5405	-303	710	76

H(18E)	5953	-252	485	76
H(18F)	4542	-300	516	76
H(19D)	4938	-181	1218	76
H(19E)	5525	24	1438	76
H(19F)	6349	-46	1239	76
H(20B)	5324	2006	1793	49
H(23B)	6369	3638	2403	48
H(24B)	6201	2568	2704	56
H(25B)	5047	885	2704	58
H(26B)	4190	280	2399	52
H(27B)	5395	500	162	53
H(28D)	7329	1224	56	91
H(28E)	7258	890	293	91
H(28F)	7193	2204	225	91
H(29C)	5545	1529	-145	50
H(29D)	4283	1757	-40	50
H(30C)	4875	3575	65	57
H(30D)	6211	3344	-8	57
H(1C)	3178	277	7402	93
H(2C)	6246	4700	6371	89
H(1C1)	5226	3657	8149	60
H(2C1)	3656	2216	8119	58
H(2C2)	4382	2524	7917	58
H(3C)	4575	476	8151	48
H(4C1)	3849	-483	7870	60
H(4C2)	2991	591	7903	60
H(5C)	3810	1558	7627	53
H(6C)	5471	-294	7565	44
H(7C)	5483	1921	7378	39
H(8C1)	4042	1160	7177	59
H(8C2)	4658	-83	7172	59
H(9C1)	5169	1762	6916	59
H(9C2)	5636	471	6890	59
H(10C)	6763	2323	7095	41
H(12E)	7592	2270	7440	52
H(12F)	8548	1281	7399	52
H(13E)	8023	1364	7753	58
H(13F)	7719	138	7659	58
H(14C)	6020	1920	7731	41
H(16E)	6703	896	8244	56
H(16F)	7670	1094	8069	56
H(17E)	6840	2891	7991	70
H(17F)	7189	2848	8228	70
H(18G)	8004	-484	7216	78
H(18H)	6661	-725	7147	78
H(18I)	7021	-804	7383	78
H(19G)	7130	-791	7955	88
H(19H)	5848	-1073	7862	88
H(19I)	6009	-937	8104	88
H(20C)	4785	1006	8470	59
H(23C)	5049	2645	9119	60
H(24C)	4446	1558	9403	66
H(25C)	3569	-228	9348	62
H(26C)	3344	-881	9017	59
H(27C)	7907	443	6895	50
H(28G)	8916	2558	7029	93
H(28H)	9591	1732	6872	93
H(28I)	9307	1303	7099	93
H(29E)	8169	1620	6603	51
H(29F)	6790	1376	6645	51
H(30E)	6552	3225	6786	56
H(30F)	7944	3471	6760	56

Table S7d. Anisotropic displacement parameters ($Å^2$ x 10^3) for PYTLUD. The anisotropic displacement factor exponent takes the form: $-2 \pi 2 [h^2 a^{*2} U_{11} + ... + 2 h k a^* b^* U_{12}]$

	U_{11}	U_{22}	U_{33}	U_{23}	U_{13}	U_{12}
O(1A)	50(1)	88(2)	63(2)	-11(2)	-1(1)	30(1)
O(2A)	68(1)	44(1)	49(2)	-4(1)	-6(1)	-12(1)
O(3A)	45(1)	59(2)	61(2)	4(2)	9(1)	-11(1)
N(1A)	53(2)	45(2)	42(2)	-5(2)	1(2)	8(1)
N(2A)	76(2)	56(2)	50(2)	-3(2)	-3(2)	21(2)
N(3A)	65(2)	62(2)	47(2)	-2(2)	3(2)	14(2)
N(4A)	59(2)	42(2)	45(2)	-6(2)	3(2)	-3(2)
C(1A)	67(2)	51(2)	39(2)	-16(2)	-7(2)	12(2)
C(2A)	43(2)	55(2)	42(2)	-15(2)	0(2)	1(2)
C(3A)	40(2)	45(2)	46(2)	-14(2)	4(2)	-2(2)
C(4A)	50(2)	42(2)	51(2)	-12(2)	13(2)	1(2)
C(5A)	34(2)	50(2)	52(2)	-7(2)	-2(2)	10(2)
C(6A)	37(2)	38(2)	45(2)	-10(2)	-1(2)	-3(2)
C(7A)	38(2)	34(2)	37(2)	-3(2)	0(2)	0(1)
C(8A)	40(2)	50(2)	49(2)	-3(2)	-3(2)	6(2)
C(9A)	56(2)	55(2)	46(2)	-7(2)	-12(2)	10(2)
C(10A)	41(2)	38(2)	39(2)	-2(2)	-4(2)	2(2)
C(11A)	37(2)	40(2)	34(2)	-9(2)	-1(2)	0(2)
C(12A)	38(2)	67(2)	40(2)	-11(2)	-3(2)	13(2)
C(13A)	40(2)	83(3)	40(2)	-16(2)	-4(2)	9(2)
C(14A)	37(2)	55(2)	36(2)	-10(2)	-1(2)	3(2)
C(15A)	40(2)	65(2)	37(2)	-16(2)	1(2)	-6(2)
C(16A)	43(2)	118(4)	41(2)	-18(3)	-3(2)	14(2)
C(17A)	66(2)	91(3)	45(2)	-15(2)	-7(2)	39(2)
C(18A)	53(2)	56(2)	55(2)	-12(2)	11(2)	-10(2)
C(19A)	70(2)	99(3)	61(3)	-34(2)	18(2)	-41(2)
C(20A)	54(2)	41(2)	48(2)	-6(2)	1(2)	8(2)
C(21A)	45(2)	40(2)	39(2)	0(2)	5(2)	-3(2)
C(22A)	43(2)	42(2)	35(2)	-2(2)	6(2)	-11(2)
C(23A)	55(2)	46(2)	44(2)	-1(2)	0(2)	-11(2)
C(24A)	68(2)	51(2)	35(2)	0(2)	-3(2)	-23(2)
C(25A)	75(2)	44(2)	48(3)	-12(2)	11(2)	-11(2)
C(26A)	77(2)	47(2)	51(3)	-6(2)	7(2)	1(2)
C(27A)	56(2)	58(2)	39(2)	-10(2)	-6(2)	-4(2)
C(28A)	46(2)	109(3)	44(2)	-25(2)	5(2)	4(2)
C(29A)	55(2)	56(2)	56(3)	3(2)	1(2)	-7(2)
C(30A)	54(2)	54(2)	57(3)	6(2)	-18(2)	-13(2)
C(31A)	36(2)	41(2)	61(3)	2(2)	-2(2)	0(2)
O(1B)	33(1)	78(2)	42(1)	-6(1)	2(1)	-10(1)
O(2B)	85(2)	43(1)	48(2)	-2(1)	-11(1)	-5(1)
O(3B)	133(2)	55(2)	34(2)	-10(1)	-7(2)	7(2)
N(1B)	52(2)	30(2)	34(2)	-2(2)	0(1)	-6(1)
N(2B)	72(2)	40(2)	37(2)	-3(2)	0(2)	-8(1)
N(3B)	72(2)	39(2)	39(2)	-2(2)	-4(2)	-5(1)
N(4B)	39(1)	36(2)	37(2)	2(1)	0(1)	2(1)
C(1B)	62(2)	38(2)	29(2)	8(2)	-1(2)	-5(2)
C(2B)	45(2)	47(2)	34(2)	1(2)	2(2)	4(2)
C(3B)	36(2)	38(2)	34(2)	9(2)	0(1)	-1(1)

C(4B)	38(2)	53(2)	34(2)	2(2)	6(2)	-2(2)
C(5B)	30(2)	45(2)	40(2)	-1(2)	0(1)	-4(2)
C(6B)	35(2)	36(2)	39(2)	2(2)	1(1)	6(1)
C(7B)	36(2)	32(2)	36(2)	-1(2)	0(1)	3(1)
C(8B)	40(2)	55(2)	37(2)	-1(2)	-1(2)	6(2)
C(9B)	46(2)	51(2)	43(2)	-3(2)	-2(2)	5(2)
C(10B)	42(2)	40(2)	32(2)	1(2)	-1(2)	6(2)
C(11B)	39(2)	39(2)	37(2)	-2(2)	-1(2)	3(2)
C(12B)	39(2)	65(2)	37(2)	-1(2)	3(2)	2(2)
C(13B)	36(2)	64(2)	36(2)	-2(2)	2(2)	-1(2)
C(14B)	35(2)	38(2)	34(2)	0(2)	2(1)	1(1)
C(15B)	32(2)	39(2)	35(2)	3(2)	1(1)	3(1)
C(16B)	37(2)	62(2)	39(2)	-3(2)	2(2)	-3(2)
C(17B)	50(2)	59(2)	40(2)	-3(2)	2(2)	-20(2)
C(18B)	57(2)	47(2)	48(2)	-1(2)	3(2)	14(2)
C(19B)	57(2)	48(2)	48(2)	1(2)	1(2)	12(2)
C(20B)	60(2)	28(2)	33(2)	2(2)	1(2)	-8(2)
C(21B)	30(2)	31(2)	41(2)	-1(2)	0(2)	1(1)
C(22B)	30(2)	35(2)	32(2)	-2(2)	4(2)	7(2)
C(23B)	39(2)	41(2)	40(2)	-4(2)	-1(2)	5(2)
C(24B)	54(2)	47(2)	40(2)	-7(2)	-2(2)	13(2)
C(25B)	58(2)	51(2)	35(2)	9(2)	8(2)	16(2)
C(26B)	43(2)	42(2)	45(2)	12(2)	10(2)	6(2)
C(27B)	51(2)	45(2)	37(2)	-4(2)	5(2)	8(2)
C(28B)	55(2)	87(3)	41(2)	3(2)	6(2)	20(2)
C(29B)	49(2)	47(2)	31(2)	-6(2)	0(2)	6(2)
C(30B)	59(2)	49(2)	34(2)	-2(2)	3(2)	4(2)
C(31B)	44(2)	48(2)	44(2)	-1(2)	6(2)	-1(2)
O(1C)	41(1)	93(2)	52(2)	-6(2)	-4(1)	-30(1)
O(2C)	86(2)	50(1)	42(2)	11(1)	7(1)	18(1)
O(3C)	82(2)	81(2)	39(2)	3(2)	11(1)	31(2)
N(1C)	62(2)	31(2)	37(2)	-2(2)	2(1)	-1(1)
N(2C)	54(2)	36(2)	46(2)	-1(2)	-4(2)	-1(1)
N(3C)	47(2)	40(2)	48(2)	-3(2)	-3(1)	-3(1)
N(4C)	44(1)	42(2)	36(2)	-2(2)	-1(1)	-3(1)
C(1C)	79(2)	32(2)	39(2)	8(2)	-2(2)	-1(2)
C(2C)	51(2)	54(2)	40(2)	5(2)	-1(2)	16(2)
C(3C)	34(2)	45(2)	41(2)	6(2)	-2(2)	-3(2)
C(4C)	36(2)	66(2)	49(2)	1(2)	2(2)	-16(2)
C(5C)	35(2)	55(2)	41(2)	0(2)	-7(2)	-16(2)
C(6C)	34(2)	34(2)	43(2)	2(2)	-2(2)	-4(1)
C(7C)	31(2)	34(2)	34(2)	3(2)	-6(1)	-1(1)
C(8C)	37(2)	65(2)	45(2)	3(2)	-7(2)	-6(2)
C(9C)	45(2)	63(2)	40(2)	7(2)	-8(2)	-6(2)
C(10C)	35(2)	31(2)	38(2)	1(2)	-4(2)	4(1)
C(11C)	30(2)	34(2)	35(2)	2(2)	-8(1)	2(1)
C(12C)	31(2)	66(2)	33(2)	2(2)	1(1)	-4(2)
C(13C)	38(2)	69(2)	37(2)	3(2)	-5(2)	-7(2)
C(14C)	31(2)	37(2)	35(2)	5(2)	-2(1)	-4(1)
C(15C)	36(2)	38(2)	37(2)	5(2)	-5(2)	1(1)
C(16C)	39(2)	66(2)	36(2)	9(2)	-3(2)	-3(2)
C(17C)	64(2)	69(3)	41(2)	-2(2)	3(2)	-27(2)
C(18C)	55(2)	48(2)	53(2)	10(2)	5(2)	11(2)
C(19C)	77(2)	49(2)	49(2)	10(2)	3(2)	13(2)
C(20C)	71(2)	30(2)	45(2)	1(2)	7(2)	-3(2)
C(21C)	36(2)	39(2)	39(2)	0(2)	-1(2)	-1(2)
C(22C)	34(2)	38(2)	40(2)	-8(2)	-1(2)	2(2)
C(23C)	61(2)	42(2)	47(2)	-8(2)	-4(2)	-4(2)
C(24C)	64(2)	62(2)	38(2)	-7(2)	-2(2)	-5(2)
C(25C)	59(2)	66(3)	31(2)	4(2)	2(2)	-3(2)
C(26C)	57(2)	44(2)	46(2)	0(2)	4(2)	-8(2)
C(27C)	53(2)	38(2)	35(2)	3(2)	3(2)	9(2)
C(28C)	32(2)	104(3)	50(2)	12(2)	1(2)	4(2)
C(29C)	43(2)	44(2)	41(2)	0(2)	4(2)	5(2)
C(30C)	56(2)	46(2)	36(2)	4(2)	-6(2)	2(2)
C(31C)	41(2)	41(2)	41(2)	0(2)	1(2)	1(2)

Table S7e. Hydrogen-bonds for PYTLUD [Å and deg.].

D-H...A	d(D-H)	d(H...A)	d(D...A)	<(DHA)
O(1A)-H(1A)...N(3A)#1	0.83	2.69	3.377(4)	141.1
O(2A)-H(2A)...N(4B)	0.83	1.87	2.682(3)	166.0
O(1B)-H(1B)...N(3C)#2	0.83	2.28	3.079(3)	162.2
O(2B)-H(2B)...N(4A)#3	0.83	1.85	2.667(3)	167.0
O(2C)-H(2C)...N(4C)#4	0.83	1.84	2.661(3)	171.6

Symmetry transformations used to generate equivalent atoms:
#1 x+1/2,-y-1/2,-z #2 x-1/2,-y+1/2,-z+1 #3 x,y+1,z
#4 -x+1,y+1/2,-z+3/2

UV-Vis and Emission spectra

Figure S6. UV-Vis absorption spectrum (blue) and room temperature emission spectrum (red) in acetonitrile of [Ir(**1**)$_2$(pytl-Me)]Cl (λ_{exc}= 335 nm). 77K emission spectrum (green) in glassy butyronitrile matrix of [Ir(**1**)$_2$(pytl-Me)]Cl (λ_{exc}= 335 nm).

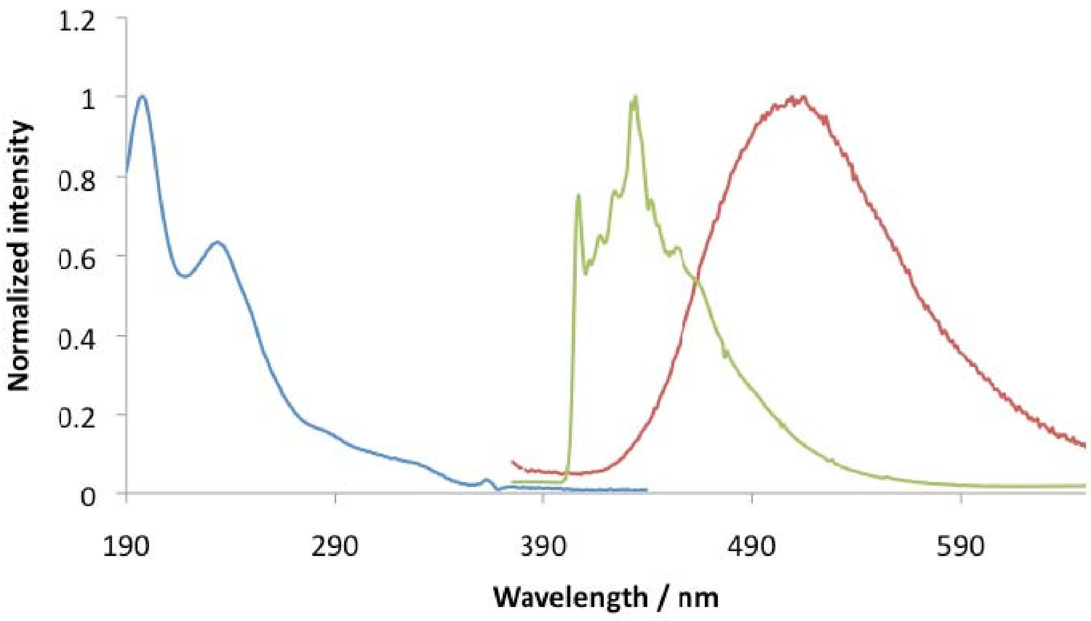

Figure S7. UV-Vis absorption spectrum (blue) and room temperature emission spectrum (red) in acetonitrile of [Ir(**2**)$_2$(pytl-ada)]Cl (λ_{exc}= 335 nm). 77K emission spectrum (green) in glassy butyronitrile matrix of [Ir(**2**)$_2$(pytl-ada)]Cl (λ_{exc}= 335 nm).

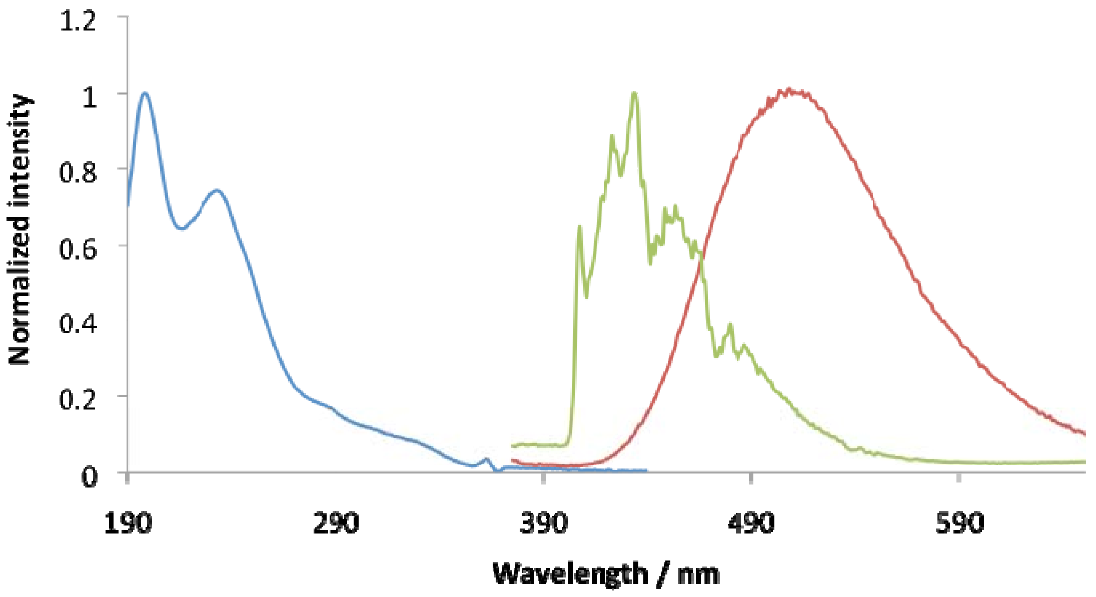

Figure S8. UV-Vis absorption spectrum (blue) and room temperature emission spectrum (red) in acetonitrile of [Ir(**1**)(**2**)(pytl-ada)]Cl (λ_{exc}= 335 nm). 77K emission spectrum (green) in glassy butyronitrile matrix of [Ir(**1**)(**2**)(pytl-ada)]Cl (λ_{exc}= 335 nm).

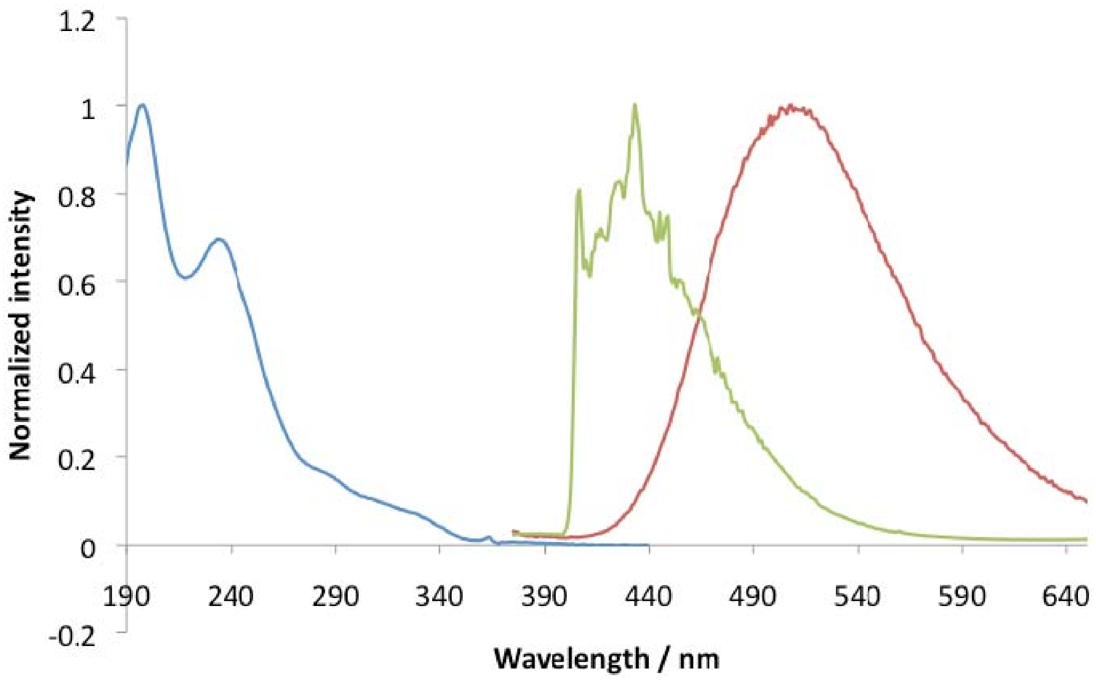

Figure S9. UV-Vis absorption spectrum (blue) and room temperature emission spectrum (red) in acetonitrile of [Ir(**1**)$_2$(pytl-DC)]Cl (λ_{exc}= 335 nm). 77K emission spectrum (green) in glassy butyronitrile matrix of [Ir(**1**)$_2$(pytl-DC)]Cl (λ_{exc}= 335 nm).

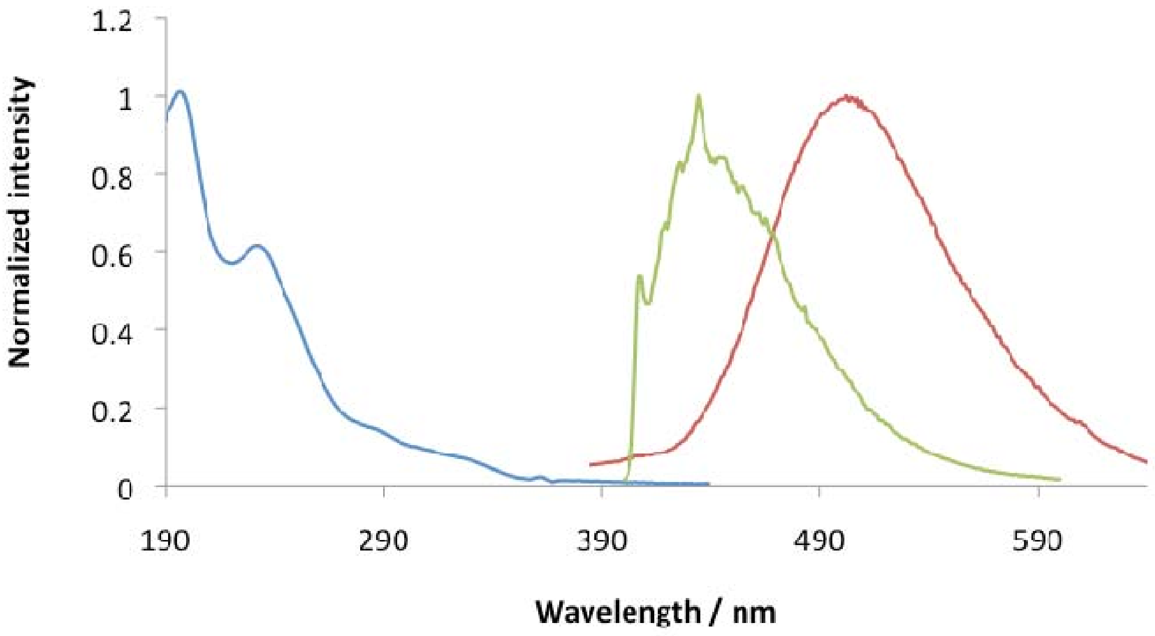

Figure S10. UV-Vis absorption spectrum (blue) and room temperature emission spectrum (red) in acetonitrile of [Ir(**1**)$_2$(pytl-βCD)]Cl (λ_{exc}= 335 nm). 77K emission spectrum (green) in glassy butyronitrile matrix of [Ir(**1**)$_2$(pytl- βCD)]Cl (λ_{exc}= 335 nm).

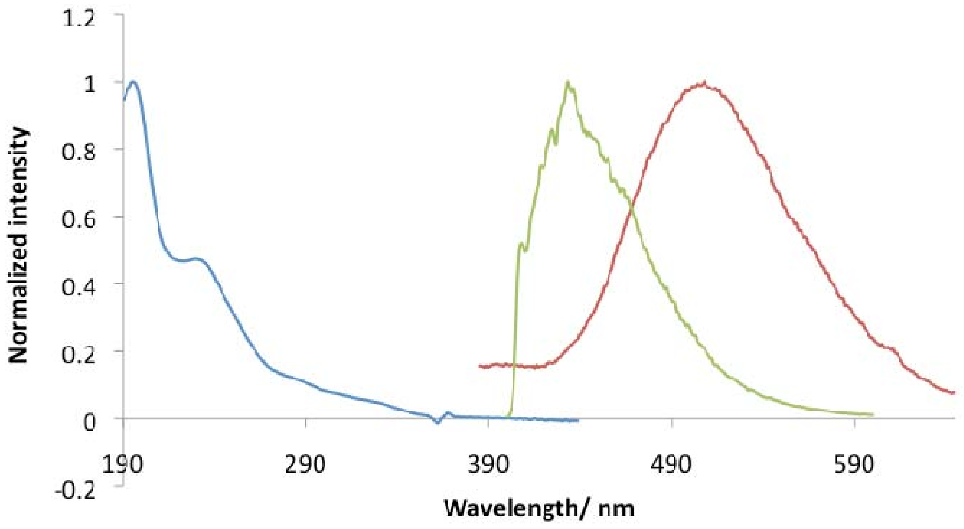

1. Sheldrick, G.M. *SHELXS. Acta Cryst. A46* **1990**, 467-473.
2. de Gelder, R.; de Graaff, R.A.G.; Schenk, H. Automatic Determination of Crystal Structures Using Karle-Hauptman Matrices. *Acta Cryst.* **1993**, *A49*, 287-293.
3. Spek, A.L. *Platon. A Multipurpose Crystallographic Tool* **2003**.

4. Felici, M.; Contreras-Carballada, P.; Vida, Y.; Smits, J.M.M.; Nolte, R.J.M.; De Cola, L.; Williams, R.M.; Feiters, M.C. Ir(III) and Ru(II) Complexes Containing Triazole-Pyridine Ligands: Luminescence Enhancement upon Substitution with β-Cyclodextrin. *Chem. Eur. J.* **2009**, *15*, 13124-13134.
5. Obata, M.; Kitamura, A.; Mori, A.; Kameyama, C.; Czaplewska, J.A.; Tanaka, R.; Kinoshita, I.; Kusumoto, T.; Hashimoto, H.; Harada, M.; Mikata, Y.; Funabiki, T.; Yano, S. Syntheses, Structural Characterization and Photophysical Properties of 4-(2-Pyridyl)-1,2,3-triazole Rhenium(I) Complexes. *Dalton Trans.* **2008**, 3292-3300.
6. Alvarez, M.; Jover, A.; Carrazana, J.; Meijide, F.; Soto, V.H.; Tato, J.V. Crystal Structure of Chenodeoxycholic Acid, Ursodeoxycholic Acid and Their Two 3β,7α- and 3β,7β-dihydroxy Epimers. *Steroids* **2007**, *72*, 535-544.
7. Hofmann, A.F.; Sjövall, J.; Kurz, G.; Radominska, A.; Schteingart, C.D.; Tint, G.S.; Vlahcevic, Z.R.; Setchell, K.D.R. A Proposed Nomenclature for Bile Acids. *J. Lipid. Res.* **1992**, *33*, 599-604.
8. Sheldrick, G. M. *SADABS. Program for Emperical Absorption Correction*; University of Göttingen: Germany, 1996.
9. Sheldrick, G. M. *SHELXL-97. Program for the Refinement of Crystal Structures*; University of Göttingen: Germany, 1997.

© 2010 by the authors; licensee Molecular Diversity Preservation International, Basel, Switzerland. This article is an open-access article distributed under the terms and conditions of the Creative Commons Attribution license (http://creativecommons.org/licenses/by/3.0/).
